# FTO‐mediated m^6^A demethylation regulates IGFBP3 expression and AKT activation through IMP3‐dependent P‐body re‐localisation in lung cancer

**DOI:** 10.1002/ctm2.70392

**Published:** 2025-07-07

**Authors:** Haiyang Wang, Hui Peng, Zhenzhen Zhang, Yilimunuer Abulimiti, Jiayi Hu, Yongxin Zhou, Ping Ji, Dong Li

**Affiliations:** ^1^ Department of Laboratory Medicine Tongji Hospital of Tongji University School of Medicine, Tongji University Shanghai China; ^2^ National Institute of Parasitic Diseases, Chinese Center for Disease Control and Prevention (Chinese Center for Tropical Diseases Research) Shanghai China; ^3^ Department of Thoracic and Cardiovascular Surgery Tongji Hospital of Tongji University School of Medicine, Tongji University Shanghai China

**Keywords:** FTO, IGFBP3, lung adenocarcinoma, m^6^A, P‐bodies

## Abstract

Lung cancer remains one of the leading causes of cancer‐related deaths worldwide, and a growing body of evidence suggests that RNA modifications, including methylation, play a critical role in its progression. In this study, we investigated the role of the RNA demethylase fat mass and obesity‐associated protein (FTO) in lung cancer progression and determined the underlying molecular mechanisms. FTO expression was significantly upregulated in LUAD and correlated with poor prognosis. FTO knockdown in lung patient‐derived organoids and LUAD cell lines reduced their proliferation, invasion, and migration, and FTO knockdown in a Kras^G12D^ mouse model reduced the growth of lung tumours. Mechanistically, FTO demethylated m^6^A sites in the insulin‐like growth factor‐binding protein 3 (IGFBP3) 3′UTR, preventing IMP3 binding. The ribonuclear protein IMP3 was identified as a crucial functional reader that interacted with m^6^A‐modified sites in the IGFBP3 3′UTR, thereby promoting IGFBP3 mRNA localisation to P‐bodies and suppressing its translation. Elevated IGFBP3 activated AKT signalling and promoted tumour progression. Collectively, we revealed that FTO drives lung cancer progression via m^6^A‐dependent sequestration of IGFBP3 mRNA into P‐bodies by IMP3, which suppresses translation and activates AKT signalling. The FTO–IGFBP3–AKT axis thus represents a promising therapeutic target.

**Key points:**

- FTO regulates the translation of IGFBP3 by demethylating m6A sites in the 3′‐untranslated region of IGFBP3 mRNA.
- Binding of the m6A reader protein IMP3 to 3′UTR m6A sites in IGFBP3 mRNA promoted its localisation and sequestration in cellular organelles known as to P‐bodies, thereby suppressing IGFBP3 mRNA translation.
- IGFBP3 regulates activation of the AKT signalling pathway, and that FTO‐mediated regulation of IGFBP3 influences LUAD malignant behaviours.

## Graphical abstract

1. FTO regulates the translation of IGFBP3 by demethylating m6A sites in the 3′‐untranslated region of IGFBP3 mRNA.
2. Binding of the m6A reader protein IMP3 to 3′UTR m6A sites in IGFBP3 mRNA promoted its localisation and sequestration in cellular organelles known as to P‐bodies, thereby suppressing IGFBP3 mRNA translation.
3. IGFBP3 regulates activation of the AKT signalling pathway, and that FTO‐mediated regulation of IGFBP3 influences LUAD malignant behaviours.

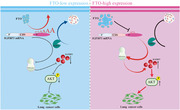

## 1 INTRODUCTION

Lung adenocarcinoma (LUAD) is the predominant subtype of non‐small cell lung cancer and is one of the leading causes of cancer‐related mortality worldwide.^1^, ^2^ Despite significant advances in diagnosis and treatment, the prognosis for LUAD patients remains poor, largely due to its high propensity for metastasis and frequent resistance to therapy. Therefore, understanding the molecular mechanisms that drive LUAD progression is essential for the development of innovative and effective therapeutic strategies.

Fat mass and obesity‐associated protein (FTO), an RNA demethylase, has emerged as a key regulator in diverse biological processes,^3^ including metabolism, immunity, and tumourigenesis.^4^, ^5^ FTO catalyses the demethylation of N6‐methyladenosine (m^6^A), the most abundant epigenetic modification in eukaryotic RNA, as well as other RNA methylations, such as N6,2′‐O‐dimethyladenosine (m^6^Am).^6^ Dysregulation of FTO has been implicated in various cancers as either a tumour suppressor or an oncogene, depending on the cellular context.^7^ In several cancers, such as acute myeloid leukaemia^8^ and glioblastoma,^9^ FTO promotes tumourigenesis by demethylating m^6^A on the transcripts of critical oncogenes; conversely, FTO acts as a tumour suppressor in other cancers, such as prostatic cancer,^10^ through its regulation of key downstream targets and signalling pathways. Although these findings highlight the potential for FTO to be a therapeutic target in cancer treatment, recent studies have indeed demonstrated that FTO promotes LUAD progression, for example by regulating autophagy,^11^ the miR‐181b‐3p/ARL5B axis,^12^ or the stability of USP7 mRNA.^13^ However, the precise molecular mechanisms, particularly those involving post‐transcriptional regulation of specific mRNA targets through m^6^A modification and their subcellular trafficking, remain incompletely understood. Crucially, whether FTO regulates LUAD progression by modulating the functional activity or spatial localisation of subcellular structures to control mRNA translation efficiency remains unknown.

The m^6^A modification plays a role at all stages of the RNA life cycle and is involved in the regulation of pre‐mRNA processing, mRNA stability, translation efficiency, and nuclear export.^14^ m^6^A is a reversible modification and is dynamically regulated by ‘writers’ (methyltransferases), ‘erasers’ (demethylases), and ‘readers’ (binding proteins), which collectively orchestrate RNA fate and function.^15^ m^6^A has pleiotropic effects on various biological processes ranging from immunity to development and stem cell biology, and has also been implicated in cancer. Thus, aberrant expression or function of enzymes involved in the m^6^A modification process, including the methyltransferases FTO, METTL3, and METTL14, and the readers YTHDF1, YTHDF2, and YTHDC, has been reported to regulate malignant progression, immune infiltration, and chemotherapy resistance through m^6^A‐mediated mechanisms in various tumours.^16^ Indeed, m^6^A modifications are increasingly recognised for their influence on tumour cell proliferation and immune evasion.^14^ While FTO‐dependent m^6^A modifications have been shown to contribute to LUAD progression by regulating various targets and pathways,^17^, ^18^, ^19^ the specific mechanisms involving the regulation of mRNA translation efficiency through the action of m^6^A readers and subsequent mRNA localisation to P‐bodies remain largely unexplored in this context.

In this study, we investigated the role of FTO in LUAD progression through an integrated analysis of clinical samples, human LUAD cell lines, and genetically engineered mice. We demonstrate that FTO is highly expressed in LUAD tissues and positively correlates with poor prognosis. Mechanistically, FTO promotes LUAD cell proliferation by demethylating m^6^A sites on transcripts of insulin‐like growth factor‐binding protein 3 (IGFBP3) a multifunctional protein involved in diverse cellular processes, resulting in enhanced IGFBP3 expression. We further show that FTO‐mediated m^6^A‐dependent regulation of IGFBP3 expression leads to activation of the AKT signalling pathway and modulates key malignant behaviours involved in cancer progression. These findings underscore the critical role of the FTO–m^6^A–IGFBP3–AKT axis in LUAD and highlight several potential targets for the development of therapeutic interventions.

## 2 RESULTS

### 2.1 High FTO expression is associated with LUAD malignancy and poor prognosis

**FIGURE 1:**
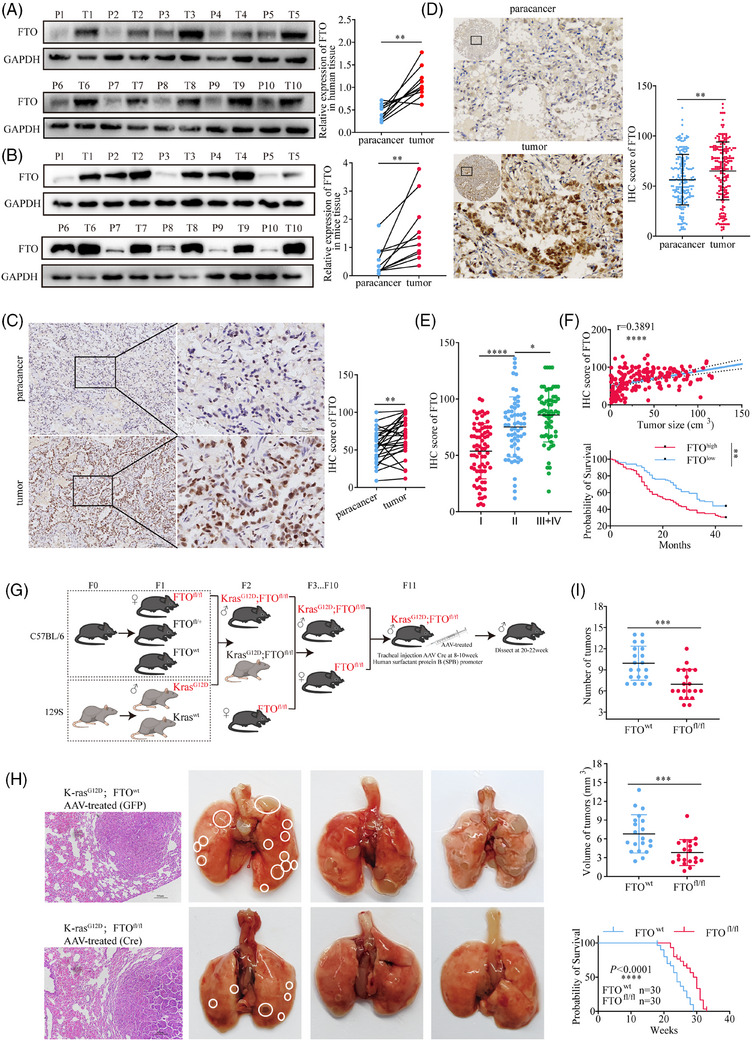
High FTO expression is associated with LUAD malignancy and poor prognosis. **(A, B)** Western blot analysis of FTO protein levels in paired tumour and adjacent non‐tumorous tissues from 10 clinical lung adenocarcinoma patients (*p*<.01, *n* = 10) and 10 Kras^G12D^ spontaneous lung cancer mice (*p*<.01, *n* = 10). **(C)** Immunohistochemistry (IHC) analysis of FTO expression in 26 paired tumour and adjacent non‐tumorous tissues from lung adenocarcinoma patient (*p*<.01, *n* = 26). **(D)** IHC staining of a tissue microarray containing samples from 196 clinical lung adenocarcinoma patients. FTO expression was significantly higher in tumour tissues compared to adjacent tissues (*p*<.01, *n* = 196). **(E)** Analysis of FTO expression in a tissue microarray, stratified by TNM staging. FTO expression progressively increased from stage I to II (*p*<.0001)and from stage II to stages III+IV (*p*<.05). **(F)** Correlation between FTO expression levels and tumour volume in lung adenocarcinoma patients (*r* = .3891, *p* < .0001). Kaplan–Meier survival analysis of FTO‐high vs. ‐low groups in 196 lung adenocarcinoma patients. Patients with higher FTO had significantly poorer survival outcomes. Log‐rank test, *p* < .01. (G) Schematic showing the creation of primary lung cancer mice with alveolar epithelial cell‐specific FTO gene knockdown (FTO‐kd). (H, I) Tumour number (*p*<.001, *n* = 20) and total tumour volume (*p*<.001, *n* = 20) were significantly reduced in FTO‐kd mice compared to wild‐type mice. Data are presented as mean ± SD, *n* = 20. Survival curve showing that FTO‐kd mice exhibited significantly longer survival compared to wild‐type mice. Log‐rank test (*p* < .0001, *n* = 30).

To assess the potential role of the FTO gene in tumourigenesis and progression, we analysed its expression across 19 types of human malignancies using data from the TCGA and GSE databases. FTO mRNA was significantly upregulated in 11 cancer types, downregulated in 4 cancer types, and showed no significant change in the remaining 4 types (Figure S1A). These observations were confirmed at the protein level by western blot analysis of FTO in 10 pairs of LUAD and adjacent non‐tumorous clinical specimens and 10 pairs of lung tumour and non‐tumour tissues from mice harbouring the G12D *KRAS* mutation, which is commonly found in human LUAD. This analysis confirmed that FTO was expressed at significantly higher levels in both human and mouse lung cancer tissues than in adjacent normal tissues (Figure 1A and B). Finally, we substantiated these findings by immunohistochemical (IHC) detection of FTO in 26 independent pairs of LUAD clinical tissues, and the results were consistent with the observations from the western blotting (Figure 1C).

We next investigated the relationship between FTO expression and LUAD progression by IHC staining of a tissue microarray of 196 LUAD specimens from patients at various disease stages. The results confirmed that FTO expression was significantly higher in tumour tissues compared with adjacent non‐tumour tissues (Figure 1D) and additionally revealed that FTO expression was positively associated with advancing TNM stage (Figure 1E) and with tumour volume (*r* = .3891) (Figure 1F). Stratification of patients into high‐ and low‐FTO expression groups further revealed a significant association between high FTO expression and poorer survival outcomes (Figure 1F). These findings strongly suggest that FTO may play a critical role in the development and progression of human lung cancer.

To confirm these findings and additionally validate the Kras^G12D^ mouse model of lung cancer for mechanistic investigations, we generated Kras^G12D^; FTO^fl/fl^ mice, in which FTO was specifically deleted in alveolar epithelial cells (Figures 1G and S1B and C). Kras^G12D^; FTO^fl/fl^ mice not only had significantly fewer and smaller lung tumours but also lived longer than control Kras^G12D^; FTO^wt^ mice (Figure 1H and I). Collectively, these results provide further evidence that FTO may play a critical role in the onset and progression of LUAD.

### 2.2 FTO plays a role in the malignant behaviour of LUAD

**FIGURE 2:**
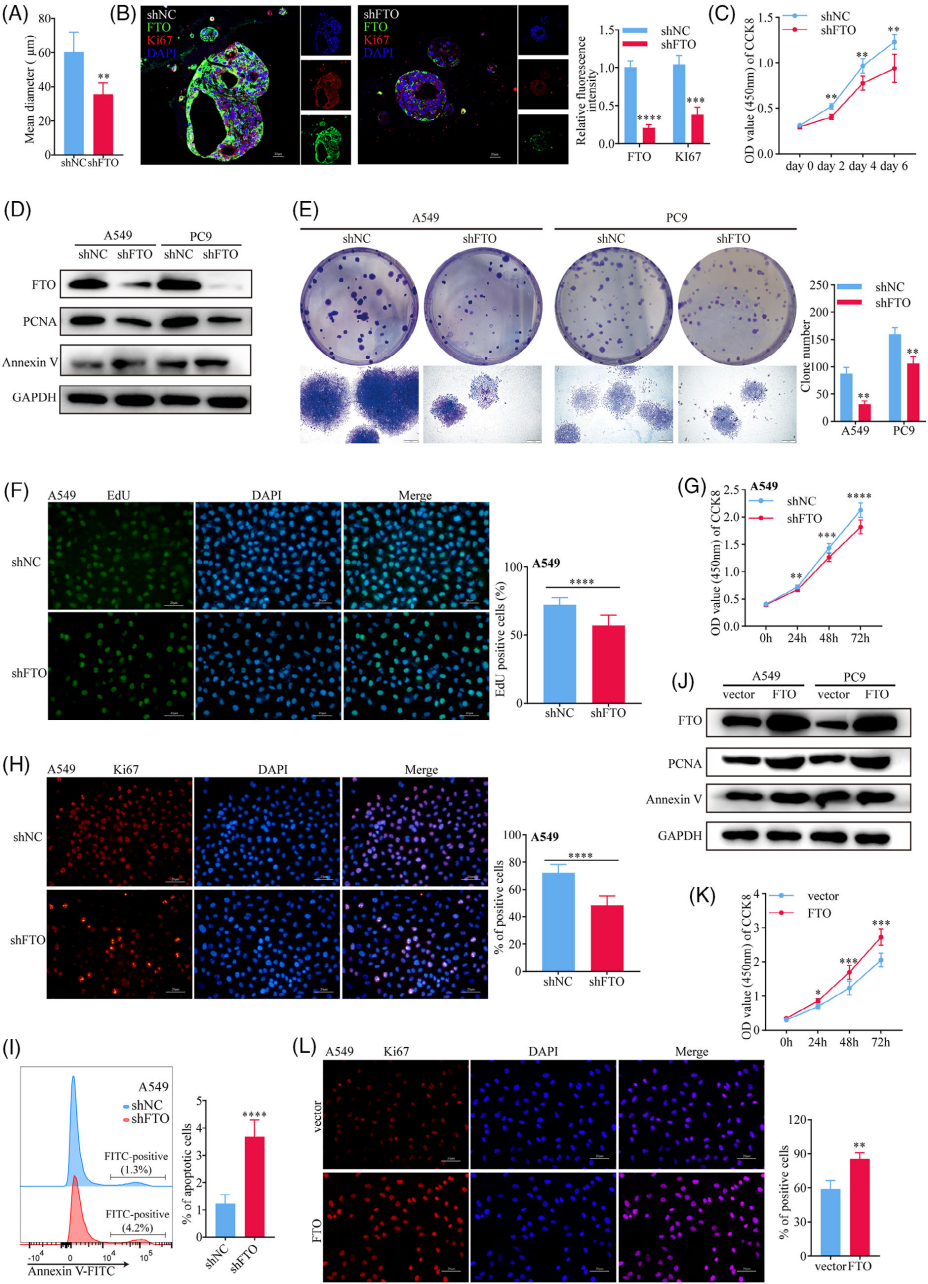
FTO plays a role in the malignant behaviour of LUAD. (A) FTO‐kd inhibited the proliferation of patient‐derived lung cancer organoids compared to shNC (*p*<.01, *n* = 3). (B) Immunofluorescence analysis showed a significant decrease in the expression levels of proliferation markers (Ki67 (*p*<.001, *n* = 3) in FTO‐kd organoids. (C) CCK8 analysis showed a significant decreased proliferative capacity of FTO‐kd organoids compared to shNC (*p*<.01, *n* = 3). (D) FTO knockdown A549 and PC9 cell lines were generated using lentivirus‐mediated shRNA. (E) Clonogenic assays demonstrated that FTO‐kd significantly inhibited the clonogenic proliferation of A549 (*p*<.01, *n* = 6) and PC9 (*p*<.01, *n* = 6) cells. (F, G) EdU (*p*<.0001, *n* = 10) and CCK8 (*p*<.0001, *n* = 10) assays further confirmed the marked reduction in the proliferative capacity of FTO‐kd A549 cells. (H, D) Western blot and immunofluorescence analysis showed a significant decrease in the expression levels of proliferation markers (Ki67 (*p*<.0001, *n* = 3) and PCNA) in FTO‐kd A549 cells. (I) Apoptosis assays revealed a significant increase in apoptotic rates of FTO‐kd A549 cells (*p*<.0001, *n* = 6). (J) Western blot analysis showed that FTO‐overexpressing (FTO‐oe) A549 and PC9 cell lines were successfully established. (K, L) CCK8 assays (*p*<.001, *n* = 10) and Ki67 immunostaining (*p*<.01, *n* = 3) demonstrated that FTO‐oe significantly enhanced the proliferation of A549 cells.

Firstly, we successfully established patient‐derived lung cancer organoid models (Figure S2A) and performed FTO gene knockdown using a constructed shRNA lentiviral vector. The expression of green fluorescent protein (GFP) indicated stable transfection efficiency mediated (Figure S2B). Haematoxylin and eosin (H&E) staining confirmed the histological characteristics of lung adenocarcinoma (Figure S2C). On day 28 post‐transfection, we measured the organoid diameters to evaluate their size. The results demonstrated that FTO knockdown (FTO‐kd) significantly inhibited organoid growth compared to the control group (shNC) (Figure 2A). Immunofluorescence analysis (IF) further revealed that FTO‐kd notably reduced the expression of Ki67 (Figure 2B), a marker of cellular proliferation. Consistently, CCK‐8 assay results corroborated these findings (Figure 2C).

To further investigate the cellular functions of FTO in lung cancer, we transduced the human LUAD cell lines A549 and PC9 with lentiviruses encoding negative control (NC) or FTO‐specific shRNA (Figures 2D and S2D). Notably, both the clonogenic potential (as measured by colony‐forming assays) and proliferative capabilities (EdU and CCK8 assays) of A549 and/or PC9 cell lines was specifically reduced by FTO‐kd (Figures 2E‐G and S2E and F). Consistent with these findings, immunofluorescence and western blot analysis of the proliferation markers Ki67 and PCNA showed a significant reduction in the FTO‐kd cells (Figures 2G and H and S2G), whereas apoptosis was significantly increased in both FTO‐kd A549 (Figure 2D and I) and PC9 (Figures 2D and S2H) cell lines compared with the corresponding shNC lines. In addition, we observed a notable decrease in the invasion (Figure S2I) and migration (Figure S2J) capabilities of FTO‐kd compared with control A549 and PC9 cells. Similar experiments performed with FTO‐overexpressing (FTO‐oe) A549 and PC9 cell lines (Figure 2J) demonstrated a significant increase in proliferation, as measured by CCK8 assays (Figure 2K) and Ki67 staining (Figure 2L), following FTO overexpression. Taken together, these in vitro results are consistent with the in vivo observations and support a role for FTO in promoting malignant behaviours associated with lung cancer progression.

### 2.3 FTO regulates expression of IGFBP3 mRNA in an m^6^A‐dependent manner

**FIGURE 3:**
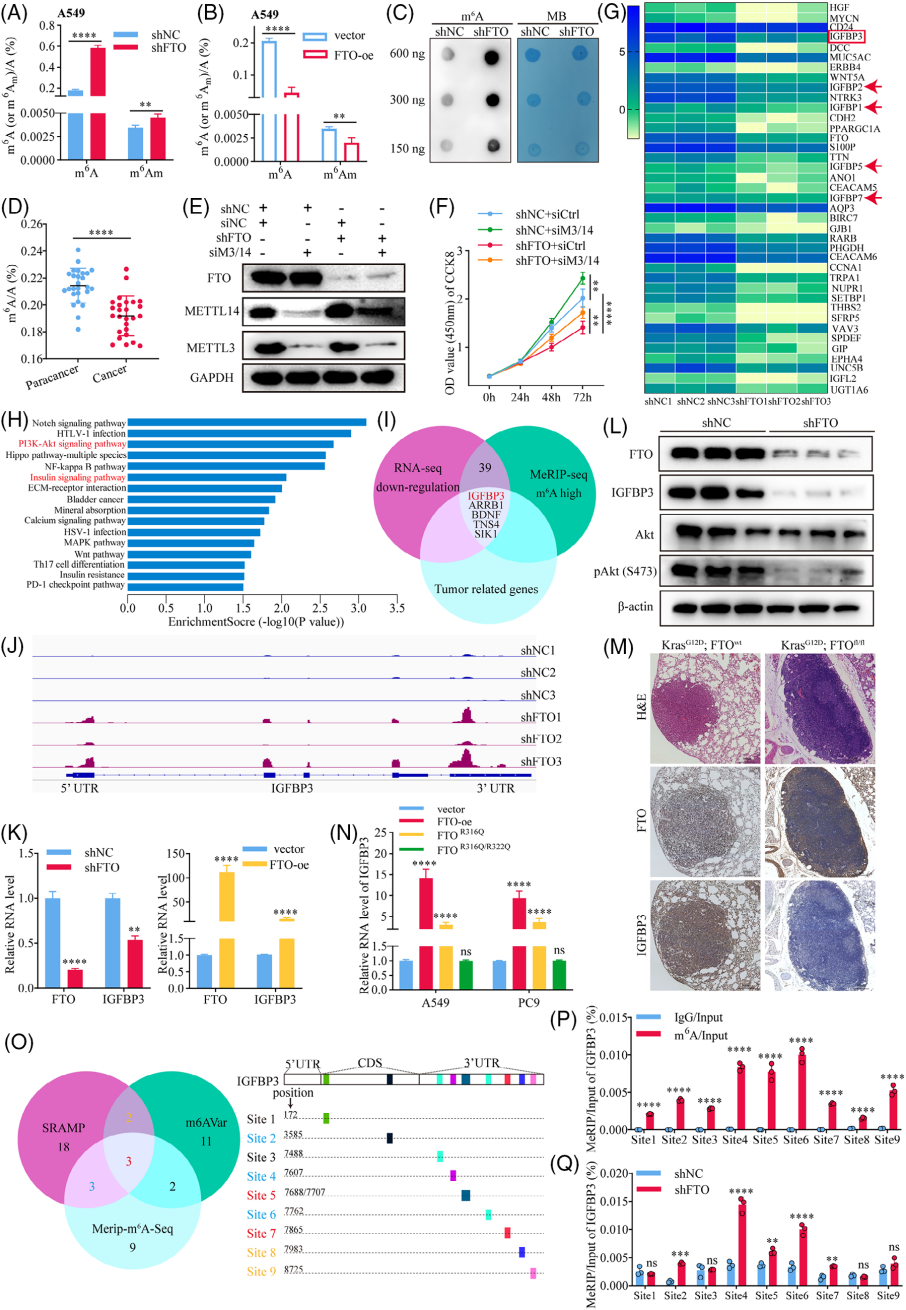
FTO regulates expression of IGFBP3 mRNA in an m^6^A‐dependent manner. (A, B) Quantification of m^6^A and m^6^Am levels in FTO‐kd and FTO‐oe A549 cells. FTO‐kd significantly increased m^6^A (*p*<.0001, *n* = 3) and m^6^Am (*p*<.01, *n* = 3) levels, while FTO‐oe decreased their levels (*p*<.0001, *n* = 3), (*p*<.01, *n* = 3). (C) Dot blots analysis confirmed the increased m^6^A in FTO‐kd A549 cells. (D) ELISA‐based quantification of m^6^A levels in RNA from lung cancer patient tissues. Total m^6^A levels were significantly lower in tumour tissues compared to adjacent tissues (*p*<.0001, *n* = 26). (E, F) Knockdown of m^6^A methyltransferases (Mettl3 and Mettl14) rescued the reduced proliferation caused by FTO‐kd, as shown by CCK8 assays (*p*<.0001, *n* = 10). (G) Heat map analysis of differentially expressed genes based on RNA‐seq in FTO‐kd A549 cells vs. control cells (*n* = 3). (H) KEGG pathway analysis of RNA‐seq data identified significant enrichment in the insulin signalling pathway in FTO‐kd A549 cells. (I) Integration of RNA‐seq and MeRIP‐seq data identified IGFBP3 as a key downstream target of FTO. (J) Integrative Genomics Viewer (IGV) visualisation of m^6^A modification sites on IGFBP3 mRNA showed increased m^6^A enrichment near the 3′UTR in FTO‐kd cells. (K, L) qPCR and Western blot analyses confirmed that IGFBP3 mRNA and protein levels were significantly reduced in FTO‐kd cells (*n* = 3). (M) IHC analysis of lung tissues from Kras^G12D^; FTO^wt^ and Kras^G12D^; FTO^fl/fl^ mice demonstrated that FTO negatively regulates IGFBP3 protein expression. (N) Cells ectopically expressing wild‐type FTO or FTO mutants with partial (R316Q) or complete (R316Q/R322Q) loss of catalytic activity showed that FTO's regulation of IGFBP3 expression depends on its m^6^A demethylase activity (*n* = 3). (O) Identification of 10 potential m^6^A sites on the IGFBP3 transcript using SRAMP, m^6^AVar, and MeRIP‐seq data. (P, Q) MeRIP‐qPCR confirmed that both above ten sites existed m^6^A, and sites 2, 4, 5, 6, and 7 were regulated by FTO. FTO‐kd increased m^6^A at these sites (*n* = 3).

We next sought to unravel some of the downstream events that mediate FTO functions in lung cancer. Because FTO is known to demethylate both m^6^A and m^6^Am modifications,^20^, ^21^ we quantified total m^6^A and m^6^Am in FTO‐kd and FTO‐oe A549 and PC9 cell lines and found that both modifications were significantly more abundant in FTO‐kd cells (Figures 3A and S3A) and, conversely, were substantially decreased in FTO‐oe cells (Figures 3B and S3B) compared with the corresponding shNC cell lines. Independent m^6^A dot blot assays also demonstrated a substantial increase of m^6^A in FTO‐kd compared with shNC A549 cells (Figure 3C). These results confirm the role of FTO in modulating these epigenetic marks in the LUAD cell lines. We also performed nucleic acid modification mass spectrometry to quantify changes in m^6^A, m^6^Am, and an additional three key RNA epigenetic modifications,^22^ namely, N1‐methyladenosine (m^1^A), 5‐methylcytidine (m^5^C), and 7‐methylguanosine (m^7^G) in FTO‐kd and FTO‐oe cells. FTO significantly modulated the levels of each modification except m^5^C, with m^6^A showing the most pronounced changes (Figure S3C). To determine whether FTO regulates LUAD behaviour through its m^6^A demethylase activity, we quantified the proportion of m^6^A of total RNA in 26 paired tissues from lung cancer patients using an ELISA. Indeed, the percentage of m^6^A‐modified RNA was significantly lower in tumour tissues compared with adjacent non‐tumour tissues (Figure 3D), whereas m^6^Am levels were not significantly different (Figure S3D); these results are consistent with an m^6^A‐specific and ‐dependent role for FTO in lung cancer proliferation. We tested this possibility by simultaneous siRNA‐mediated knockdown of Mettl3 and Mettl14^23^ (Figure 3E). Notably, Mettl3/Mettl14‐kd enhanced cell proliferation compared with shNC cells, which contrasted with the effect of FTO‐kd, and concomitant knockdown of FTO and Mettl3/Mettl14 rescued the proliferation defect of FTO‐kd cells (Figure 3F), consistent with the opposing effects of these enzymes on m^6^A modification. These results indicate that FTO regulates LUAD proliferation is mediated via an m^6^A‐dependent mechanism.

We next investigated the potential downstream targets of FTO by performing RNA‐seq analysis of FTO‐kd and shNC A549 cells. This analysis identified 656 and 495 genes that were significantly upregulated and downregulated, respectively, by FTO knockdown (Figure S3E). Because FTO are known to negatively regulate m^6^A sites,^24^ we focused on investigating the demethylated gene sequences (Figure 3G). Notably, they included several members of the insulin‐like growth factor (IGF)‐binding protein (IGFBP) family, including IGFBP3, IGFBP2, IGFBP1, IGFBP5, and IGFBP7 (Figure 3G). KEGG pathway analysis further revealed that the components of the insulin signalling pathway were significantly enriched in FTO‐kd A549 cells compared with shNC cells (Figure 3H). Therefore, to determine whether FTO activity might regulate insulin signalling in LUAD cells via alterations in m^6^A, we performed MeRIP‐seq by immunoprecipitating m^6^A from cell total RNAs and sequencing the m^6^A‐pulldown RNAs. This analysis identified 450 hypomethylated mRNAs and 701 hypermethylated mRNAs, respectively, in FTO‐kd compared with control A549 cells (Figure S3F). MeRIP‐seq identified 8660 m^6^A peaks common to both shNC and FTO‐kd A549 cells, with 2908 and 6103 peaks being unique to the control and FTO‐kd A549 cells, respectively (Figure S3G). Among these, 1021 and 2304 m^6^A sites were hypomethylated and hypermethylated, respectively, by FTO knockdown (Figure S3H). Sequencing of the m^6^A‐modified RNA revealed that most m^6^A peaks were located in the coding sequence (CDS) and 3′ untranslated regions (3′UTRs) of the transcripts (Figure S3I), with the density being highest in the 3′UTR (Figure S3J). This finding is consistent with previous reports^16^ that FTO‐kd significantly increases m^6^A levels in the 3′UTR of mRNAs. Representative motif analysis identified DDGACU and DDACDA (D = A/G/U) as key motifs in transcripts from both FTO‐kd and shNC cells (Figure S3K). To identify the potential mRNAs regulated by FTO via modulation of m^6^A in LUAD cells, we integrated the RNA‐seq and MeRIP‐seq data and found 39 potential target genes. We focused on mRNAs potentially involved in lung cancer progression through extensive literature review^25^, ^26^, ^27^, ^28^ and finally identified five mRNAs as potential FTO targets (Figure 3I). Based on KEGG pathway and differential gene analysis (Figure 3G and H), we hypothesised that IGFBP3 may be a key downstream target of FTO. To validate this, we used Integrative Genomics Viewer^29^ to compare the sequencing depth between the MeRIP and input samples from FTO‐kd and shNC groups, visualising m^6^A modification sites on IGFBP3 mRNA (Figure 3J). The results showed that FTO knockdown did indeed increase the m^6^A content of IGFBP3 mRNA, with particular enrichment near the 3′UTR (Figure S3I and J). This suggested that IGFBP3 mRNA is likely regulated by FTO through m^6^A.

In addition, IGFBP3 protein and mRNA expression decreased and increased in parallel with FTO in FTO‐kd and FTO‐oe A549 cells, respectively (Figure 3K and L). IHC analysis of lung tissues from the Kras^G12D^ mouse model confirmed that expression of IGFBP3 decreased in Kras^G12D^; FTO^fl/fl^ mice compared with control mice (Figure 3M), demonstrating that FTO positively regulates IGFBP3 protein expression. To determine whether FTO‐mediated regulation of IGFBP3 depends on its m^6^A catalytic activity, we compared IGFBP3 transcript levels in A549 cells overexpressing FTO wild‐type protein or mutant FTO proteins with partial (FTO^R316Q^) or complete (FTO ^R316Q/R322Q^) loss of catalytic activity^30^ (Figure S3L). Indeed, IGFBP3 transcript levels were positively correlated with FTO catalytic activity, with the most striking elevation in IGFBP3 mRNA detected in FTO wild‐type‐overexpressing cells, followed by reduced levels in FTO^R316Q^ cells, and levels in FTO ^R316Q/R322Q^ cells that were not significantly different from those in vector control cells (Figure 3N). These results are consistent with our hypothesis that FTO regulates IGFBP3 transcript levels in an m^6^A‐dependent manner.

We next investigated the mechanism by which FTO‐mediated m^6^A demethylation regulates IGFBP3 mRNA levels. We identified potential m^6^A modification sites on IGFBP3 mRNA using SRAMP and m^6^AVar databases^31^, ^32^ and compared the results with our MeRIP‐seq data (Figure 3O). This analysis identified 10 potential m^6^A modification sites (since adenines 7688 and 7707 are too close to be distinguished, they are combined into site 5) in IGFBP3 mRNA, which was confirmed by MeRIP‐qPCR (Figure 3P). MeRIP‐qPCR analysis of FTO‐kd cells demonstrated that m^6^A modification at sites 2, 4, 5, 6, and 7 were significantly enriched by FTO knockdown (Figure 3Q) and significantly reduced by FTO overexpression (Figure S3M). Moreover, the suppression of m^6^A was not seen in cells overexpressing catalytically inactive FTO^R316Q/R322Q^ (Figure S3M), indicating that the effects of FTO on m^6^A modification at these sites was directly dependent on FTO catalytic activity. Taken together, these findings identify IGFBP3 mRNA as a key downstream target of FTO in LUAD cells, and indicate that FTO‐mediated regulation of IGFBP3 expression is critically dependent on demethylation of specific m^6^A sites, primarily in the 3′UTR of IGFBP3.

### 2.4 IGFBP3 acts as a functional downstream gene of FTO through regulating the activation of AKT pathway

**FIGURE 4:**
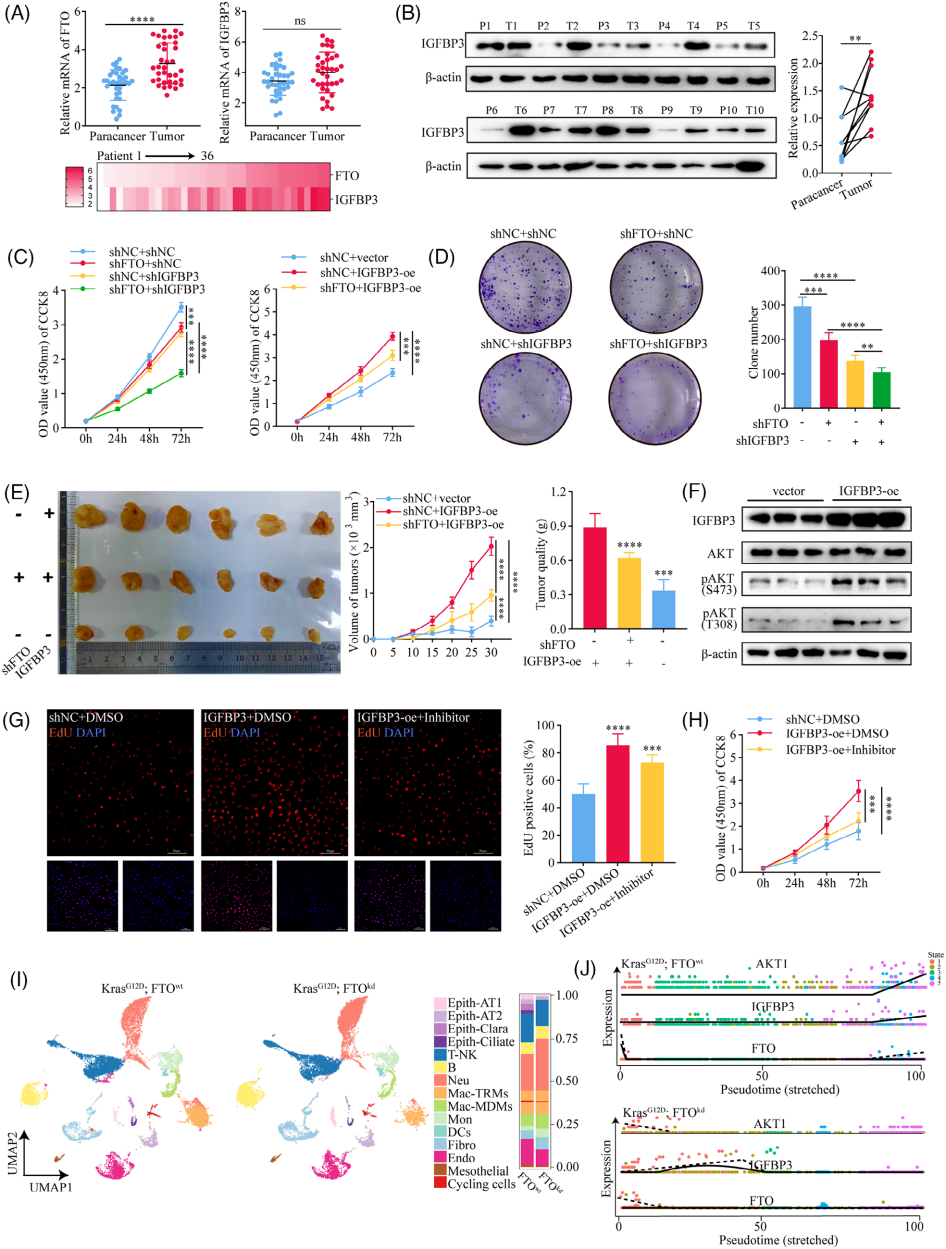
IGFBP3 acts as a functional downstream gene of FTO through regulating the activation of AKT pathway. (A) qPCR analysis of FTO (*p*<.0001, *n* = 36) and IGFBP3 (*p* = .0545, *n* = 36) mRNA expression in tumour and adjacent tissues from 36 lung adenocarcinoma patients. Heatmap analysis showed a positive correlation between FTO and IGFBP3 transcript levels (*r* = .6392, *p*<.0001). (B) Western blot analysis confirmed a significant increase in IGFBP3 protein levels in tumour tissues compared to adjacent tissues (*p*<.01, *n* = 10). (C, D) CCK8 (*p*<.0001, *n* = 10) and colony formation (*p*<.0001, *n* = 6) assays demonstrated that combined knockdown of FTO and IGFBP3 resulted in a more pronounced reduction in cell proliferation compared to FTO‐kd or IGFBP3‐kd alone. IGFBP3‐oe rescued the proliferation defect caused by FTO‐kd. (E) In vivo subcutaneous tumour models using FTO‐kd, IGFBP3‐kd, and FTO‐kd + IGFBP3‐oe cells demonstrated that IGFBP3‐oe reversed the reduction in tumour growth caused by FTO‐kd (*p*<.001, *n* = 6). (F) Western blot analysis showed that IGFBP3‐oe enhanced AKT phosphorylation at S473 and T308 (*n* = 3). (G, H) EdU (*p*<.0001, *n* = 6) and CCK8 assays (*p*<.0001, *n* = 10) demonstrated that IGFBP3‐oe promoted cell proliferation, while the AKT inhibitor Capivasertib (10 nM) partially reversed this effect. (I) UMAP‐based dimensionality reduction analysis after scRNA‐sequencing (*n* = 2) between Kras^G12D^; FTO^wt^ and Kras^G12D^; FTO^kd^. (J) Pseudotime trajectory analysis showed the expression of IGFBP3 and AKT genes along the temporal dimension.

To investigate the role of FTO‐mediated regulation of IGFBP3 in lung cancer progression, we first compared FTO and IGFBP3 transcript levels in paired tumour and adjacent normal tissues from 36 LUAD patients. FTO and IGFBP3 mRNA levels were both higher in tumour tissues than normal tissues, although the increase in IGFBP3 was not statistically significant (Figure 4A). Heatmap analysis revealed a significant positive correlation between FTO and IGFBP3 mRNA levels (*r* = .4503; Figure 4A) and this was confirmed by western blot analysis, which showed significantly elevated IGFBP3 protein levels in tumour tissues compared with adjacent normal lung tissues (Figure 4B).

Next, we explored the functional role of IGFBP3 in LUAD growth by generating A549 cell lines with FTO‐kd and concomitant IGFBP3‐kd or IGFBP3‐oe (Figure S4A and B). Combined knockdown of FTO and IGFBP3 led to a more pronounced reduction in colony formation and cell proliferation compared with knockdown of either FTO or IGFBP3 alone, and the proliferation defect caused by FTO‐kd was rescued by IGFBP3‐oe (Figure 4C and D). To validate these findings in vivo, we established a mouse model of subcutaneous LUAD by injection of A549 cells with IGFBP3‐oe alone or FTO‐kd and IGFBP3‐oe in nude mice, and measured tumour volumes every 5 days (Figure 4E). The in vivo data confirmed our in vitro results, with IGFBP3‐oe reversing the inhibition of tumour growth observed in FTO‐kd cells.

IGFBP3 has both intracellular and extracellular functions,^33^ and previous studies have shown that IGFBP3 expression is closely associated with activation of the PI3K–AKT signalling pathway.^34^, ^35^ Therefore, we examined the potential involvement of IGFBP3 in regulating this pathway in LUAD cells. Analysis of differential gene expression in SAS cells with and without IGFBP3‐kd from GSE205275 database, followed by KEGG pathway and gene set enrichment analyses revealed significant enrichment of components of the PI3K‐AKT pathway in IGFBP3‐kd cells (Figure S4C–E). AKT expression was notably downregulated (Figure S4F), consistent with our previous RNA‐seq analysis upon FTO‐kd cells, which also showed PI3K‐AKT pathway enrichment (Figure 3H). In keeping with a possible role for IGFBP3–AKT signalling in FTO function, we found that IGFBP3‐oe enhanced AKT phosphorylation at S473 and T308 (Figure 4F), indicative of AKT activation, and promoted cell proliferation (Figure 4G and H). The latter effect was partially reduced by incubation of IGFBP3‐oe cells with the Akt inhibitor capivasertib (10 nM) (Figure 4G and H), confirming a requirement for AKT. These findings support a role for the FTO–m^6^A–IGFBP3–AKT axis in promoting LUAD cell proliferation.

IGFBP3 can exert its biological functions both intracellularly via shuttling between the nucleus and cytoplasm, and extracellularly via secretion and modulation of IGF‐dependent effects.^36^, ^37^ To determine whether IGFBP3 activates AKT in LUAD cells via an extracellular, IGF‐dependent pathway, we examined p‐AKT levels in A549 cells treated with conditioned medium collected from IGFBP3‐oe cells. Although IGFBP3 concentrations were elevated in supernatants from IGFBP3‐oe cells compared with control cells (165.4 ng/mL vs. 37.86 ng/mL; Figure S4G), AKT was not activated by addition of conditioned medium (Figure S4H) or by increasing the concentration to 100 nM by supplementation with purified exogenous IGFBP3 (Figure S4I). Similarly, treatment of IGFBP3‐oe cells with an IGF1/2R inhibitor (NVP‐NEW541, 200 nM) did not block activation of AKT (Figure S4J). These results confirmed that IGFBP3 does not activate AKT in LUAD cells through IGF‐dependent signalling but likely does so through an intracellular mechanism.

Type II alveolar epithelial cells (AT2 cells) are known to serve as critical progenitor cells for LUAD, and both cell types show some overlap in gene expression patterns, particularly in surfactant‐related genes (e.g., SFTPC) and developmental genes (e.g., NKX2‐1).^38^, ^39^ To further investigate the functional regulation of IGFBP3 and the AKT pathway by FTO, we conducted scRNA‐seq analysis of lung tumours from Kras^G12D^; FTO^kd^ and Kras^G12D^ control mice. UMAP‐based dimensionality reduction analysis revealed a significant increase in epithelial‐like tumour cells in the lungs of Kras^G12D^ mice compared with Kras^G12D^; FTO^kd^ mice (Figure 4I). Pseudotime trajectory analysis showed a marked upregulation of IGFBP3 and AKT genes along the temporal dimension in Kras^G12D^; FTO^wt^ mice, while the opposite trend was observed in Kras^G12D^; FTO^kd^ mice (Figure 4J). Taken together, these findings support the hypothesis that FTO‐ and m^6^A‐mediated regulation of IGFBP3 leads to activation of the AKT pathway through an IGF‐independent mechanism, thereby contributing to lung cancer progression.

### 2.5 IMP3 inhibits the translation efficiency of IGFBP3 mRNA in an m^6^A‐dependent manner

**FIGURE 5:**
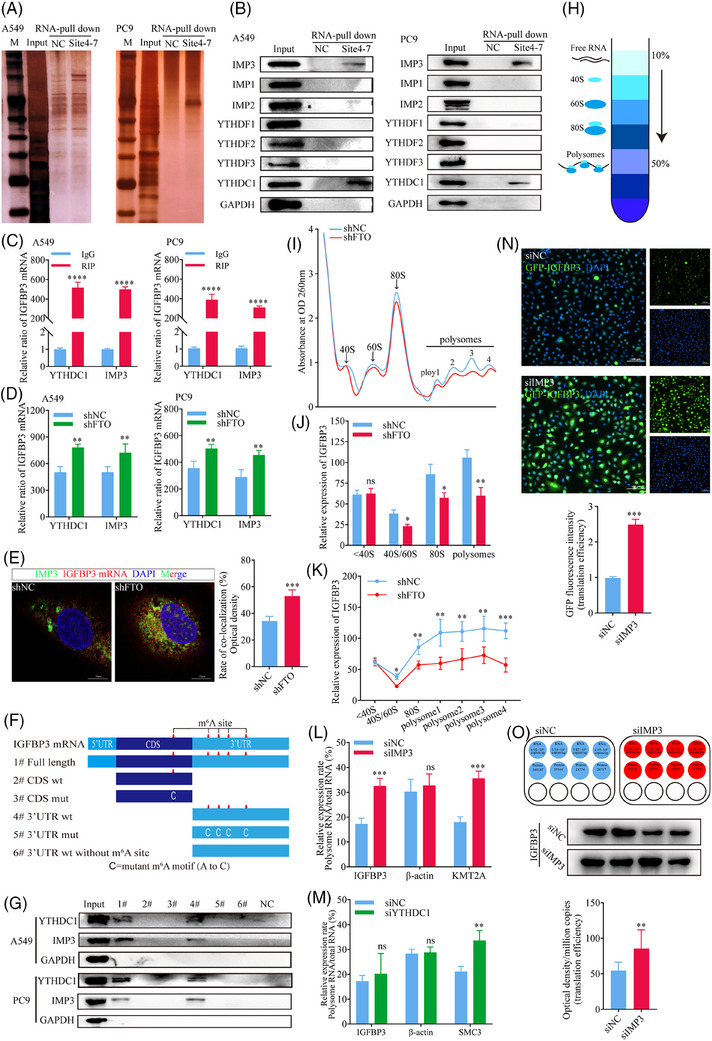
IMP3 inhibits the translation efficiency of IGFBP3 mRNA in an m^6^A‐dependent manner. (A) Products of RNA‐pulldown experiments with biotinylated RNA targeting m^6^A positions 4–7 were analysed by silver staining. (B) Western blot analysis after RNA‐pulldown assays using biotinylated RNA probes targeting m^6^A sites 4–7 of IGFBP3 showed that YTHDC1 and IMP3 bind to these sites in A549 and PC9 cells. (C) qPCR after RNA Binding Protein Immunoprecipitation (RIP) assays with anti‐YTHDC1 and anti‐IMP3 antibodies confirmed their binding to m^6^A‐modified sites in the 3′UTR of IGFBP3 mRNA (*n* = 3). (D) RIP‐qPCR analysis showed that FTO‐kd significantly increased the binding of YTHDC1 and IMP3 to IGFBP3 mRNA in an m^6^A‐dependent manner. (E) The immunofluorescence co‐localisation analysis indicated that FTO‐kd enhance the co‐localisation signals of IMP3 and IGFBP3 (*p*<.001, *n* = 3). (F, G) RNA‐pulldown assays using six biotinylated RNA fragments of IGFBP3 revealed that YTHDC1 and IMP3 bind specifically to the wild‐type 3′UTR but not to mutated or unmodified 3′UTR fragments. (H) The schematic showed the manner in which the sucrose density gradient separates polyribosomes. (I–K) Ribosome profiling showed that FTO‐kd decreased the translation efficiency (TE) of IGFBP3 mRNA (*n* = 3). (L, M) TE analysis of IGFBP3 following knockdown of IMP3 or YTHDC1. IMP3‐kd significantly enhanced IGFBP3 TE (*p*<.001, *n* = 3), while YTHDC1‐kd had no effect (*p* = .305, *n* = 3). (N) GFP‐tagged IGFBP3 plasmids transfected into IMP3‐kd and control A549 cells demonstrated that IMP3 inhibits IGFBP3 TE, as measured by GFP fluorescence (*p*<.001, *n* = 6). (O) Absolute quantification of IGFBP3 transcript copy number and TE in IMP3‐kd and control cells (*p*<.01, *n* = 4).

The functional consequences of the m^6^A RNA modification are mediated via specific m^6^A‐binding proteins (m^6^A readers) that act as potent post‐transcriptional modulators of mRNA translation efficiency and stability.^40^, ^41^ To identify proteins that might influence IGFBP3 mRNA via binding to m^6^A sites in its 3′UTR, we first examined the correlation between expression of IGFBP3 and putative m^6^A reader proteins in the TCGA database. This analysis identified a significant correlation only with the Insulin‐like growth factor 2 mRNA‐binding protein 3 (IMP3) (Figure S5A). Indeed, RNA‐pulldown assays performed by incubating A549 and PC9 cell lysates with biotinylated RNA probes targeting m^6^A sites 4 to 7 (Figure 3O) in the IGFBP3 transcript identified IMP3 and YTHDC1 as binding proteins likely to interact with m^6^A in IGFBP3 mRNA (Figure 5A and B). To confirm this, we performed RIP‐qPCR by quantifying RNA complexes pulled down from cell lysates with anti‐YTHDC1 and anti‐IMP3 antibodies. The results confirmed that both YTHDC1 and IMP3 bind to m^6^A‐modified sites in the 3′UTR of IGFBP3 (Figure 5C). Moreover, RIP‐qPCR analysis of A549 and PC9 cells based on anti‐YTHDC1 and anti‐IMP3 antibodies showed a significant increase in both YTHDC1 and IMP3 binding to IGFBP3 mRNA in FTO‐kd cells compared with NC cells (Figure 5D), suggesting that the protein–RNA interactions were m^6^A‐dependent and regulated by FTO. At the same time, we found that the elevation of m^6^A caused by FTO‐kd promoted the binding ability of IMP3 to IGFBP3 mRNA, which further suggested that the binding of IMP3 to IGFBP3 was regulated by FTO (Figure 5E). To confirm specific binding of YTHDC1 and IMP3 to IGFBP3 mRNA, we performed RNA agarose gel electrophoresis on the PCR products, which revealed a band size consistent with the expected size of 229 bp for m^6^A sites 4–7 in IGFBP3 mRNA (Figure S5B).We also examined the binding specificity of YTHDC1 and IMP3 to IGFBP3 by incubating cell lysates with biotinylated RNA fragments corresponding to various IGFBP3 mRNA sequences, namely, full‐length mRNA, the wild‐type CDS, mutated CDS (A to C), wild‐type 3′UTR, mutated 3′UTR (A to C), and wild‐type 3′UTR without the m^6^A modification (Figure 5F). RNA‐pulldown assays revealed that only the full‐length IGFBP3 mRNA and wild‐type 3′UTR probes could pull down YTHDC1 and IMP3 (Figure 5G). Taken together, these findings establish YTHDC1 and IMP3 as m^6^A readers that specifically recognise m^6^A modification sites in the 3′UTR of IGFBP3, thereby regulating IGFBP3 mRNA expression.

Because the m^6^A modification has broad effects on RNA metabolism and function, including regulation of splicing, stability, translation efficiency, degradation,^42^ secondary structure, protein binding, and translation initiation,^43^ we next examined whether FTO influences the nuclear export, stability, or translation of IGFBP3 mRNA or the stability of IGFBP3 protein. We found that FTO did not affect the stability of IGFBP3 mRNA, as shown by monitoring the decay rate of existing IGFBP3 mRNA in FTO‐kd or NC A549 and PC9 cells after incubation with actinomycin D to inhibit new RNA synthesis (Figure S5C and D). Similarly, FTO did not affect the transcription of IGFBP3, as shown by dual‐luciferase reporter assays in which luciferase expression was driven by the IGFBP3 promoter (Figure S5E and F). To investigate potential effects on IGFBP3 mRNA splicing rate, A549 cells were treated with actinomycin D and levels of pre‐mRNA and nascent transcripts were measured by qPCR. However, the IGFBP3 transcript splicing rate was not significantly different between FTO‐kd and control cells (Figure S5G and H). Moreover, separation of nuclear and cytoplasmic RNA (Figure S5I and J) revealed no differences in the subcellular localisation of IGFBP3 mRNA between FTO‐kd and control cells (Figure S5K and L), indicating that FTO does not affect the nuclear export of IGFBP3 mRNA. Finally, we investigated the stability of existing IGFBP3 protein by western blot analysis of cells treated with cycloheximide to block new protein synthesis. Consistent with the mRNA stability results, the half‐life of IGFBP3 protein was similar in FTO‐kd and control A549 cells (Figure S5M). Thus, FTO does not regulate IGFBP3 expression by affecting transcription; nuclear export, stability, or splicing of the mRNA; or stability of the protein.

We next investigated the possibility that FTO may regulate IGFBP3 expression by modulating its translation efficiency (TE) by performing ribosome profiling based on sucrose gradient centrifugation on A549 FTO‐kd and control cells (Figure 5H). After separating RNA fractions into non‐translating, translation initiation, and translation‐active polysomes (Figure 5I), we observed a significant reduction in IGFBP3 mRNA in 40S/60S, 80S, and polysomes of FTO‐kd cells compared with control cells (Figure 5J and K), suggesting that FTO may decrease the expression of IGFBP3, at least in part, by reducing its TE. We next determined whether IMP3 and YTHDC1 might play a role in altering IGFBP3 mRNA TE by performing ribosome profiling of A549 and PC9 IMP3‐kd and YTHDC1‐kd cells. The TE of KMT2A and SMC3 mRNAs, which are known to be inhibited by IMP3 and YTHDC1, respectively, were included as positive controls in the analysis.^44^, ^45^ Notably, knockdown of IMP3, but not YTHDC1, significantly enhanced IGFBP3 TE (Figures 5L and M and S5N), suggesting that IMP3 specifically recognises the m^6^A modification in the 3′UTR of IGFBP3 and inhibits its translation, whereas the function of YTHDC1 binding to IGFBP3 3′UTR remains unknown. To confirm the relationship between IGFBP3 and IMP3, we transfected IMP3‐kd and control A549 cells with a plasmid encoding GFP‐tagged IGFBP3 and examined the cells by fluorescence microscopy (Figure 5N). IMP3‐kd significantly increased IGFBP3 TE in these cells, as shown by fluorescence imaging of IGFBP3. Finally, quantification of the absolute copy number of IGFBP3 mRNA after normalisation of total RNA in each condition,^46^ western blot analysis showed that IGFBP3 TE increased significantly in IMP3‐kd cells compared with control (Figure 5O).

Collectively, these results demonstrate that IMP3 is a specific reader of m^6^A sites 4–7 in the 3′UTR of IGFBP3 and regulates its translation in an FTO‐dependent manner.

### 2.6 IMP3 inhibits IGFBP3 mRNA translation by promoting its localisation to P‐bodies

**FIGURE 6:**
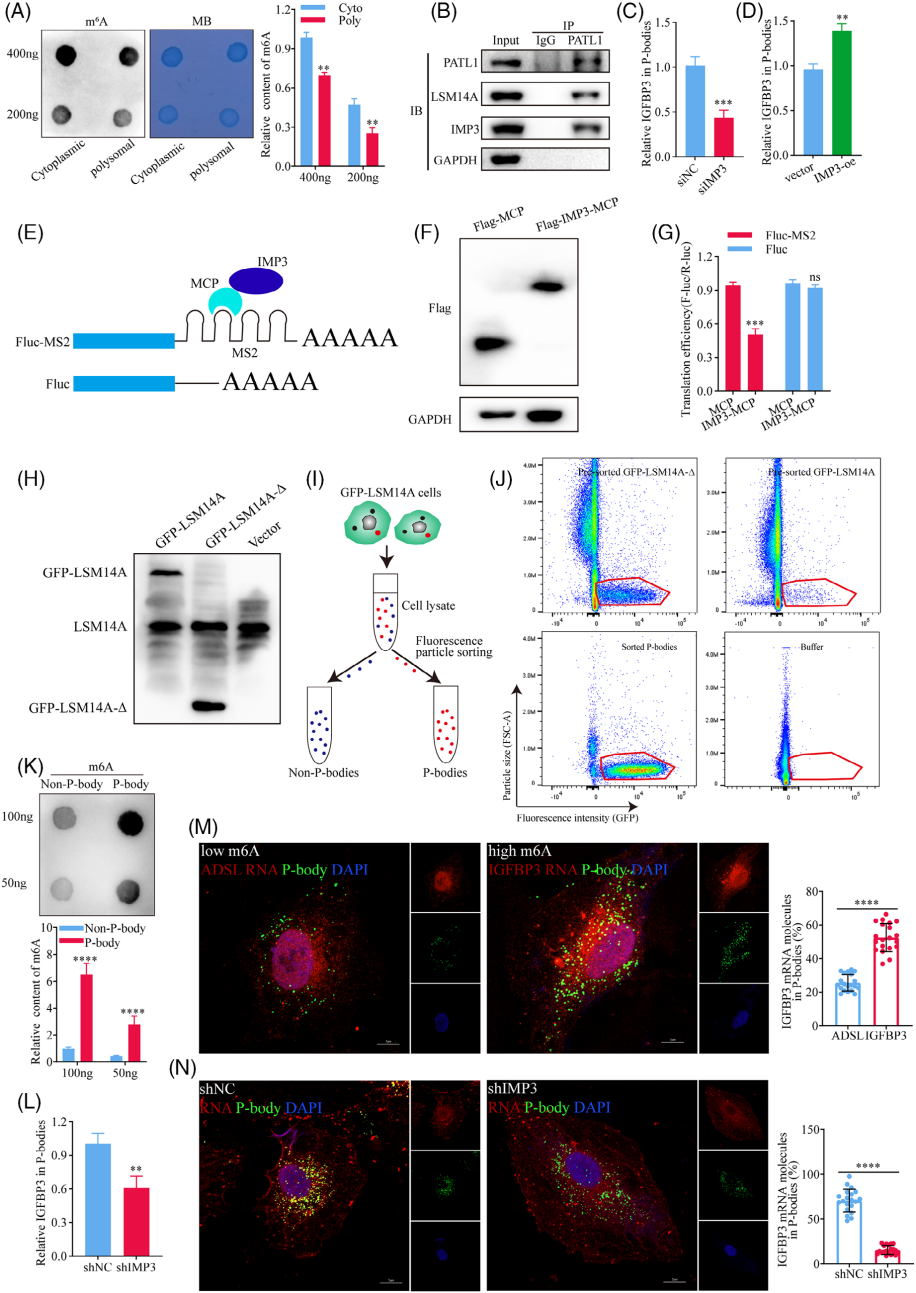
IMP3 inhibits IGFBP3 mRNA translation by promoting its localisation to P‐bodies. (A) Dot blot assay using an anti‐m^6^A antibody indicates significantly lower m^6^A modifications in polysomal fractions compared to other cytoplasmic components (*n* = 3). (B) Immunoprecipitation (IP) assay with anti‐PATL1 antibody reveals that IMP3 co‐localises with the P‐body marker LSM14A. (C) RIP‐qPCR analysis shows decreased localisation of IGFBP3 mRNA to P‐bodies upon IMP3‐kd (*p*<.001, *n* = 3), while (D) demonstrates increased P‐body localisation of IGFBP3 mRNA upon IMP3‐oe (*p*<.01, *n* = 3). (E) Schematic representation of the tethering experiment using the MCP‐IMP3 system with Rluc‐MS2 reporter mRNAs to assess IMP3's regulatory role on translation. (F) Western blot analysis confirms the expression of FLAG‐MCP (negative control) and FLAG‐IMP3‐MCP proteins, ensuring consistent transfection efficiency across samples (see Figure S6B). (G) Tethering of FLAG‐IMP3‐MCP to reporter mRNA results in a ∼45% reduction in luciferase activity (*p*<.001, *n* = 3). (H–J) Schematic representation of the method used to fluorescently label P‐bodies in the A549 cell line using GFP‐LSM14A, contrasting with a control expressing a non‐localising truncated version (GFP‐LSM14A‐Δ). Flow particle sorting (FACS) distinguishes GFP‐LSM14A‐labelled P‐bodies from non‐P‐body particles based on size and fluorescence. (K) Dot blot quantification revealed that m^6^A levels in P‐bodies are significantly higher than in non‐P‐body fractions (*n* = 3). (L) IMP3‐kd leaded to a significant reduction in IGFBP3 enrichment in P‐bodies (*p*<.01, *n* = 3). (M) FISH analysis demonstrates that IGFBP3 mRNA co‐localises with P‐bodies more frequently than ADSL mRNA, as evidenced by confocal microscopy (*p*<.0001, *n* = 3). (N) IMP3‐kd diminished the co‐localisation of IGFBP3 mRNA with P‐bodies (*p*<.0001, *n* = 3).

We next investigated the mechanisms by which IMP3 mediates translational repression of IGFBP3. Anti‐m^6^A dot blots showed that m^6^A modifications were enriched in the cytoplasmic fraction compared with polysomal fraction of A549 cells (Figure 6A), suggesting that transcripts with high m^6^A levels may have reduced ribosome‐binding capacity compared with m^6^A‐low transcripts. Unbound m^6^A‐high transcripts are known to redistribute within the cytoplasm and may associate with P‐bodies, which are membrane‐free cytoplasmic organelles involved in the turnover of mRNA molecules.^47^ Because localisation of m^6^A‐high mRNA to P‐bodies, and the subsequent reduction in TE, is influenced by m^6^A readers,^48^ we investigated whether IMP3 affects the redistribution of IGFBP3 mRNA to P‐bodies in LUAD cells. Lysates of A549 cells were immunoprecipitated with an antibody against the P‐body marker protein PATL1^49^ and subjected to western blot analysis. Notably, both the P‐body marker LSM14A^50^ and IMP3 co‐immunoprecipitated with PATL1 (Figure 6B), indicating that IMP3 likely plays a role in localisation of m^6^A‐high mRNAs to P‐bodies. It further supports the idea of Shan et al. that IMP3 can bind to m^6^A sites on target mRNAs, re‐localise them to P‐bodies, and thereby decrease the translation efficiency of these target genes. Consistent with this, RIP‐qPCR analysis of A549 cells based on IMP3 antibody showed that IGFBP3 mRNA localisation to P‐bodies was decreased in IMP3‐kd cells (Figure 6C) and increased in IMP3‐oe cells (Figure 6D) compared with the corresponding control cells. Importantly, further analysis demonstrated that IMP3‐kd did not influence the overall m^6^A content of total transcripts (Figure S6A). These results support a crucial role for IMP3 in m^6^A‐dependent localisation of IGFBP3 mRNA to P‐bodies in LUAD cells.

To provide direct evidence that IMP3 is involved in the regulation of IGFBP3 mRNA translation and localisation, we performed tethering experiments using the MS2 coat protein (MCP)‐IMP3 system with Renilla luciferase (Rluc)‐MS2 reporter mRNAs (Figure 6E). Western blot analysis confirmed the expression of FLAG‐MCP (negative control) and FLAG‐IMP3‐MCP proteins (Figure 6F). We excluded potential errors due to differences in transfection efficiency (Figure S6B). Tethering FLAG‐IMP3‐MCP to the reporter mRNA resulted in a ∼45% decrease in luciferase activity (Figure 6G) without significantly altering mRNA abundance (Figure S6B). In contrast, negative controls showed no significant changes in either protein or mRNA levels, confirming that IMP3 actively suppresses translation by redistributing its target mRNA away from ribosomes. In addition, our immunofluorescence‐based co‐localisation results showed that the co‐localisation signal between IGFBP3 mRNA and P‐bodies was enhanced after IMP3 overexpression (Figure S6C), further indicating that IMP3 plays a role in targeting IGFBP3 to P‐bodies. To further examine whether IMP3 targets IGFBP3 mRNA to P‐bodies, we used a novel method to directly purify intact P‐bodies. This was challenging due to their small size (∼500 nm) and low abundance.^51^ To address this, we utilised the enhanced sensitivity of flow particle‐sorting technology. P‐bodies were fluorescently labelled by stably expressing a canonical P‐body marker, GFP‐LSM14A, in the A549 cell line. To distinguish P‐bodies from other particles, we used a control cell line expressing a truncated GFP‐LSM14A (GFP‐LSM14A‐Δ) that does not localise to P‐bodies (Figure 6H). After cell lysis, organelle‐enriched cytosolic extracts were sorted by fluorescence‐activated cell sorting (FACS), based on size and fluorescence, allowing us to distinguish between GFP‐LSM14A‐labelled P‐bodies and non‐P‐body particles^48^, ^51^ (Figure 6I). This sorting method effectively depleted non‐P‐body particles while preserving GFP‐LSM14A‐labelled P‐bodies (Figure 6J). We found that m^6^A levels were significantly higher in P‐bodies than in the non‐P‐body fractions (Figure 6K), suggesting that P‐bodies contain a substantial amount of m^6^A‐high, non‐translating mRNA, including IGFBP3. Furthermore, IMP3‐kd significantly reduced the enrichment of IGFBP3 in P‐bodies (Figure 6L), indicating that IMP3 facilitates the localisation of IGFBP3 mRNA to P‐bodies via an m^6^A‐dependent mechanism, thereby preventing its association with ribosomes.

To further validate that IMP3 targets m^6^A‐high IGFBP3 to P‐bodies, we compared the subcellular localisation of IGFBP3 (m^6^A‐high) with ADSL (m^6^A‐low) in A549 cells based on our MeRIP‐seq data and previous reports.^52^ FISH analysis of ADSL and IGFBP3 RNA, followed by confocal microscopy, showed that a higher proportion of IGFBP3 mRNA than ADSL mRNA co‐localised with P‐bodies (Figure 6M) and this enrichment of IGFBP3 mRNA was significantly diminished by IMP3 knockdown (Figure 6N). These results indicated that hypermethylated RNA was more easily localised to P‐bodies than hypomethylated RNA and IMP3 could promote the re‐localisation of hypermethylated RNA to P‐bodies.

**FIGURE 7:**
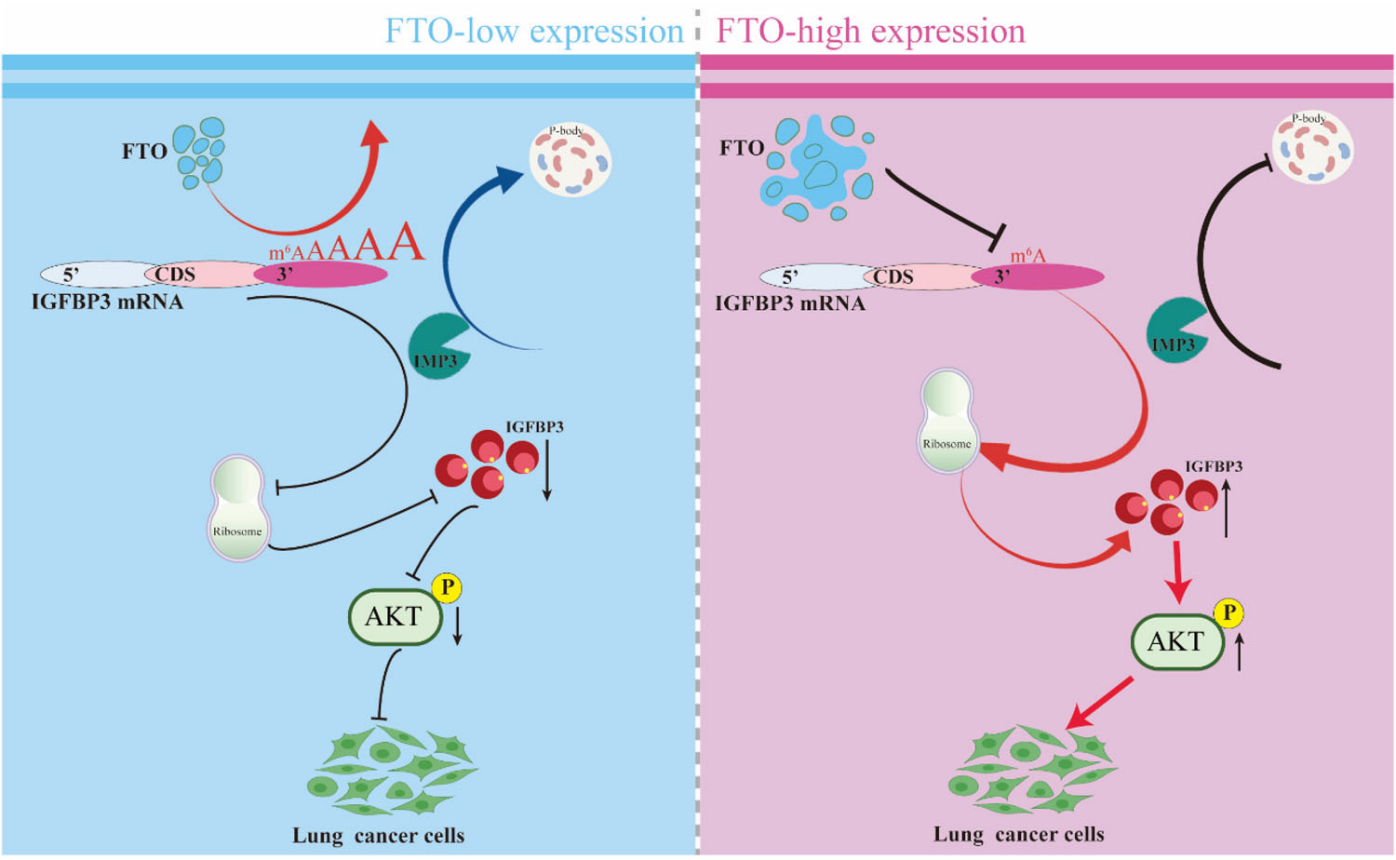
Proposed working model for promotive effects of FTO on NSCLC progression. This work finds that FTO‐mediated modulation of m^6^A and effects on IGFBP3 translation play critical oncogenic roles in LUAD. Our results suggest that targeting of the FTO–IGFBP3–AKT axis may be a promising strategy for the treatment of LUAD.

Taken together, these data demonstrate that IMP3 mediates the redistribution of m^6^A‐high IGFBP3 mRNA to P‐bodies via binding to m^6^A sites 4–7 in the 3′UTR of IGFBP3, thereby reducing its ribosomal association and TE. This process operates downstream of FTO‐mediated m^6^A regulation of IGFBP3 and highlights the functional interplay between m^6^A demethylation, mRNA localisation, and translational control (Figure 7).

## 3 DISCUSSION

FTO is well‐recognised for its involvement in human adipogenesis and obesity and has further been associated with mitochondrial biogenesis and oxidative stress through post‐transcriptional modification of relevant genes.^53^, ^54^, ^55^ FTO was the first demethylase identified that targets RNAs, including mRNA, tRNA, lncRNA, and miRNA, and has been reported to regulate dopaminergic signalling in the brain as well as mRNA splicing of critical regulatory factors involved in adipogenesis.^56^ Several studies have reported that FTO has tumour suppressor activity, and its expression is inversely correlated with the progression of ovarian cancer, hepatocellular carcinoma, intrahepatic cholangiocarcinoma, and renal cell carcinoma.^3^ Conversely, FTO may also play an oncogenic role, as demonstrated in acute myelocytic leukaemia, where FTO is expressed at elevated levels and inhibits ASB2 and RARA gene^57^ by reducing transcript m^6^A content, resulting in enhanced cell transformation and leukaemogenesis. Elevated FTO levels have also been demonstrated in cervical squamous cell carcinoma, colorectal cancer, and glioblastoma.^58^ Overall, these studies suggested that FTO may have multifunctional roles across different cancer types and tissues. Because the expression patterns and regulatory mechanisms of FTO in lung cancer are poorly understood, we undertook a comprehensive investigation of FTO expression in LUAD and the molecular mechanisms of action that contribute to disease progression.

m^6^A is the most abundant internal modification in mRNA and exerts its biological functions by recruiting m^6^A readers that regulate mRNA stability and/or TE. m^6^A has been shown to affect the RNA structure‐dependent accessibility of pre‐mRNA splicing factor heterogeneous nuclear ribonucleoprotein C (hnRNP C) protein to the introns of its targeted mRNAs,^59^ suggesting that m^6^A may act as an RNA structure switch to modulate RNA–protein interactions. Therefore, it is not surprising that m^6^A dysregulation is crucially involved in the progression of solid and non‐solid tumours, even though the underlying mechanisms differ from one malignancy to another.^52^, ^60^, ^61^ Prior studies have demonstrated that FTO promotes LUAD progression through diverse m^6^A‐dependent mechanisms, including enhancing autophagy flux by reducing m^6^A on ATG5 and ATG7 mRNA,^62^ regulating PKM2 metabolism,^63^ and influencing immune evasion.^64^

In the present study, we found that FTO‐kd in LUAD cells resulted in retention of m^6^A in the 3′UTR of IGFBP3 mRNA, leading to enhanced binding of the reader IMP3, re‐localisation to P‐bodies, and translational repression. The 3′UTR is a crucial regulatory element located downstream of the CDS in mRNA, and although not a component of the CDS, it plays a vital role in regulating mRNA stability, TE, and subcellular localisation. In cancer, 3′UTR interactions with RNA‐binding proteins and microRNAs are known to modulate tumour cell proliferation, migration, invasion, and immune evasion.^65^ Moreover, epigenetic modifications such as m^6^A in the 3′UTR play a key role in post‐transcriptional regulation^66^ by affecting mRNA stability and TE and contributing to cancer cell growth and drug resistance. Our results demonstrate that m^6^A sites in the 3′UTR of IGFBP3 play a key role in recruiting and binding IMP3, which is involved in gene regulation and modulation of tumour cell proliferation. Our study provides compelling evidence that IMP3 binding to m^6^A‐modified IGFBP3 mRNA directs it to P‐bodies, resulting in translational suppression. The precise mechanistic link within the P‐body, however, warrants further discussion. P‐bodies are dynamic hubs enriched in mRNA decay factors (e.g., DCP1/2, XRN1),^67^ translational repressors (e.g., eIF4E‐T, RCK/p54),^68^ and proteins involved in miRNA‐mediated silencing.^69^ The sequestration of IGFBP3 mRNA into these structures by IMP3 likely prevents its engagement with the translational machinery in two key ways: (1) Physical sequestration: Localisation away from ribosomes and translation initiation complexes in the cytosol inherently reduces access. (2) Recruitment of repressive machinery: IMP3 or associated factors within the P‐body may actively recruit translational repressors (e.g., interfering with 5′ cap recognition by eIF4E via eIF4E‐T)^70^ or mRNA decay enzymes.^47^ While our data show reduced IGFBP3 mRNA association with polysomes (Figure 5J and K) and increased presence in P‐bodies (Figure 6C and D and L‐N) upon m^6^A increase/IMP3 binding, future work should aim to identify the specific repressive complexes recruited to the IGFBP3 mRNP within P‐bodies upon IMP3 binding. Furthermore, it remains to be determined if the IGFBP3 mRNA localised to P‐bodies is eventually degraded or can be released and translated under certain conditions. Nevertheless, our findings establish a clear functional link between FTO‐mediated m^6^A demethylation, IMP3 reader function, P‐body localisation, and translational control of a key oncogenic signalling component in LUAD.

Given that FTO has strong demethylase activity, it is likely to have hundreds or even thousands of downstream targets; however, most are either unlikely to be involved in malignant behaviours or have expression levels functionally unaffected by FTO. This provides a key rationale for selecting IGFBP3 as a downstream functional target of FTO. IGFBP3 is primarily known for its role in binding and regulating the bioavailability of IGFs, through which it modulates cellular responses. However, our findings suggest that IGFBP3 has additional intracellular functions beyond its IGF‐binding activity.^71^ Previous studies have shown that IGFBP3 is a critical regulator of the PI3K‐Akt pathway, which is itself a central modulator of many key processes, including cell growth, survival, and metabolism.^72^ In the context of lung cancer, we observed that IGFBP3 overexpression enhanced the activity of AKT, as shown by elevated phosphorylation at S473 and T308. Thus, IGFBP3 acts as a functional downstream target of FTO in regulating AKT activity in lung cancer. Of note, our results indicate that this IGFBP3 regulatory role appears to occur in an IGF‐independent mechanism, which is consistent with an earlier report suggesting that IGFBP3 can engage intracellular signalling pathways independently of IGF binding, possibly through direct interactions with cellular kinases or other signalling intermediates.^73^ While IGFBP3 classically regulates IGF bioavailability, our data support an IGF‐independent AKT activation mechanism. IGFBP3 contains a nuclear localisation signal (NLS) and functional heparin‐binding domain (HBD) that facilitate direct interactions with PI3K regulatory subunits.^74^ It may also scaffold AKT‐PDK1 complexes at the plasma membrane.^75^ Notably, IGFBP3 can bind integrin β1 (ITGB1) to transactivate IGF1R‐independent phosphorylation cascades,^76^ providing a plausible route for AKT activation in LUAD. The results of our study provide valuable insights into the FTO–IGFBP3–AKT signalling axis in lung cancer, highlighting its potential as a diagnostic, prognostic, and therapeutic target.

Despite these strengths, our study has several limitations. First, although we elucidated the involvement of FTO in regulating IGFBP3 through m^6^A demethylation, other downstream targets of FTO remain unexplored. Second, we primarily focused on LUAD, and the relevance of the FTO–IGFBP3 axis to other tumour types remains to be explored. Third, we did not perform in‐depth analyses of the role of other epitranscriptomic marks influenced by FTO, such as m1A or m^6^Am, which could offer additional insights into the broader regulatory mechanisms of FTO. Finally, the precise mechanisms by which IGFBP3 activates AKT, which may involve direct binding to PI3K–Akt pathway components or other regulatory proteins, remain to be elucidated.

## 4 CONCLUSION

This study demonstrated that upregulated FTO in LUAD regulates the translation of IGFBP3 by demethylating m^6^A sites in the 3′‐untranslated region of IGFBP3 mRNA. Binding of the m^6^A reader protein IMP3 to 3′UTR m^6^A sites in IGFBP3 mRNA promoted its localisation and sequestration in cellular organelles known as to P‐bodies, thereby suppressing IGFBP3 mRNA translation. Loss‐ and gain‐of‐function assays showed that IGFBP3 expression regulates activation of the central AKT signalling pathway, and that FTO‐mediated regulation of IGFBP3 influences LUAD malignant behaviours in vitro and lung tumour growth in a KrasG12D mouse model. Taken together, our results uncover a novel role for FTO in promoting LUAD growth through an m^6^A‐dependent pathway that involves modulation of IGFBP3 mRNA translation and AKT activation.

## 5 METHODS

### 5.1 Animals

Kras^G12D^ mice on the 129S genetic background were purchased from the Jackson Laboratory (strain ID: 129S/Sv‐Kras^tm3Tyj/J^). This strain is characterised by expression of the G12D mutant KRAS protein, which leads to the development of lung tumours and results in death of the mice at approximately 200 days (∼7 months) of age.^77^ To generate mice on a pure C57BL/6 genetic background, male 129S/Sv‐Kras^tm3Tyj/J^ mice were crossed with female C57BL/6 FTO^fl/fl^ and the Kras^G12D^ offspring were backcrossed with C57BL/6 FTO^fl/fl^ females for 10 generations. This process ultimately eliminated the 129S genetic background, yielding Kras^G12D^ mice on a pure C57BL/6 background. Ten‐week‐old mice were anesthetised and intubated for surgery. The virus, 5E11 Cre adeno‐associated virus (pAAV‐SPB‐MCS‐T2A‐EGFP), was administered to Kras^G12D^; FTO^fl/fl^ mice via tracheal intubation to generate Kras^G12D^; FTO^kd^ mice. After administration of the virus, the incision was sutured, and the mice were transferred to a warming pad for recovery. All experimental mice were bred and maintained under specific pathogen‐free conditions at the Laboratory Animal Center, Shanghai Tongji Hospital. Mice were housed in the City of Hope Animal Facility with a 12‐h light/12‐h dark cycle, temperature of ∼18–23°C, and humidity of 40%–60%. All animal experiments were approved by the Animal Ethics Committee of Shanghai Tongji Hospital (protocol number 0401‐DW‐077).

For experiments, Kras^G12D^ C57BL/6 mice of both sexes at age 20–22 weeks were used. The mice were sacrificed by intraperitoneal injection of a lethal dose of sodium pentobarbital and tumours were then excised for analysis. Tumour volume of Kras^G12D^ C57BL/6 mice was calculated using the formula *V* = 1/2×length (*L*)×width (*W*)^2^. After measurement, tumour protein and RNA were extracted as described below. For subcutaneous xenograft tumour model, freshly harvested A549 cells were suspended in a 1:1 mixture of serum‐free DMEM and Matrigel (Corning, USA, 356234) at a concentration of 5×10⁶ cells per 100 µL. Six‐week‐old male BALB/c nude mice were anesthetised with 2% isoflurane, and the right flank was sterilised with 75% ethanol. Using a 27‐gauge syringe, 100 µL of the cell suspension was injected subcutaneously into the right dorsal flank. Mice were monitored daily for tumour formation and overall health. Tumour dimensions (length *L* and width *W*) were measured every 5 days using digital callipers (Mitutoyo #500‐752), and volume (*V*) was calculated as *V* = (π/6)×*L*×*W* ^2^. Upon reaching a humane endpoint (tumour volume ≥ 1500 mm^3^), mice were euthanised by CO₂ asphyxiation. Tumours were excised, weighed, and divided for fixation in 10% neutral‐buffered formalin (24 h for histology) or flash‐freezing in liquid nitrogen for molecular analysis Tumour volume was calculated using the formula *V* = π/6×*L* (length) ×*W* (width)×*H* (height).

### 5.2 Maintenance and transfection of LUAD cell lines

The human LUAD cell lines A549 (passages 25–35) and PC9 (passages 20–30) were obtained from Chinese Academy of Sciences, Shanghai, China. Cells were used within 50 cumulative passages from the original stock to ensure phenotypic stability. All cells were maintained in DMEM supplemented with 10% FBS (Gibco, USA, A5670701) and 1% penicillin‐streptomycin (Fuheng biology, Shanghai, K002TM) at 37°C in a 5% CO_2_ atmosphere. Cells were transfected with plasmids to enable overexpression or short hairpin RNA (shRNA)‐mediated silencing of FTO, IGFBP3, IMP3 and YTHDC1 using Lipofectamine 3000 (Gibco, USA, L3000015). All shRNA or siRNA sequences are listed in the Table S1. Transfection efficiency was assessed by reverse‐transcription (RT)‐qPCR and western blot analyses as described below.

### 5.3 Patient‐derived lung cancer organoids construction and clinical samples collection

Small pieces (∼0.3‐0.6 cm^3^) of lung cancer tissues and adjacent non‐tumorous tissues were taken from surgically resected lung specimens as part of the lung cancer biobanking process at the Tongji Hospital of Tongji University (Shanghai, China) with patients’ informed consent. The research protocol was approved by the Ethics Committee of the Tongji Hospital of Tongji University. The entire experimental protocol was conducted in compliance with the guidelines.^78^ Samples were confirmed as tumour or normal tissue on the basis of histopathological assessment. The diagnosis of each case was confirmed by pathologists at Tongji Hospital of Tongji University. Human samples were separated and transported to the laboratory on ice within 1 h of removal from the patients in cold Hank's balanced salt solution (HBSS) (Gibco, USA, 14175095) with antibiotics. Samples were washed three times with cold HBSS with antibiotics and were sectioned with sterile surgical blades. Approximately one‐thirds of the sectioned samples were used to perform downstream experiments including extraction of total RNA and protein, and the rest were incubated with 0.001% DNase (Thermo, USA, EN0521), 1 mg/mL I collagenase/dispase (Thermo, USA, 17018029), 200 U/mL penicillin, 200 mg/mL streptomycin in DMEM/F12 medium (Gibco, USA, 21041025) at 37°C for 1 h with intermittent agitation. After incubation, the suspensions were repeatedly triturated by pipetting and passed through 70 µm cell strainers (BD, USA). The strained cells were centrifuged at 450 × *g* for 3 min at 4°C, and the pellet was resuspended in 100 µL MBM (serum‐free medium; DMEM/F12; Lonza) supplemented with 20 ng/mL of bFGF (Invitrogen, USA, 13256‐029), 50 ng/mL human EGF (Invitrogen, USA, AF‐100‐15), N2 (Invitrogen, USA, 14‐7219‐95), B27 (Invitrogen, USA, MHCIFG05), 10 µM ROCK inhibitor (Thermo, USA, A2644501), and 1% penicillin/streptomycin. One hundred microliters Matrigel (Corning, USA) was added to the remaining 50 µL suspension for establishing organoids, and the resulting cell suspension was allowed to solidify on pre‐warmed six‐well culture plates (Corning, USA) at 37°C for 30–40 min. After gelation, 3 mL MBM was added to the well. The medium was changed every 4 days, and the organoids were passaged after 1–3 weeks. For FTO knockdown, organoids were transduced with concentrated lentiviral particles encoding shFTO or shNC (MOI = 30) in the presence of 8 µg/mL polybrene. Organoids were incubated with virus‐containing medium for 48 h with gentle shaking, followed by extensive washing and fresh medium changes. Transduction efficiency was monitored by GFP fluorescence. Successful target gene knockdown was confirmed 7 days post‐transduction by qRT‐PCR analysis on lysates from pooled organoids. For passaging, a solidified Matrigel drop containing the organoids was harvested using cold DPBS and then centrifuged at 450 × *g* for 3 min at 4°C. The pellets were washed with cold DPBS and centrifuged at 250 rcf for 15 min at 4°C. The organoids were resuspended in 2 mL trypsin (Invitrogen) and incubated for 10 min at 37°C for dissociation. Afterwards, 10 mL DMEM/F12 containing 10% FBS was added and centrifuged at 450 × *g* for 3 min. The pellets were washed with DPBS and centrifuged at 450 × *g* for 3 min. The pellets were resuspended in MBM + Matrigel (Gibco, USA, A1413202) (1:3) and reseeded at 1:3–1:4 ratios to allow the formation of new organoids.

### 5.4 Polysome profiling

Polysome profiling was conducted as described previously.^79^ In brief, control (negative control shRNA [shNC]) or FTO‐kd cells from ten 10‐cm culture dishes were incubated with 100 µg/mL cycloheximide for 5 min at 37°C. The culture medium was then removed, the cells were washed with cold PBS containing 100 µg/mL cycloheximide, and lysis buffer (10 mM Tris, pH 7.4, 150 mM KCl, 5 mM MgCl_2_, 100 µg/mL cycloheximide, 0.5% Triton X‐100, protease inhibitors, and RNase inhibitor) was added at 300 µL per dish on ice for 15 min with pipetting. The cell lysate was centrifuged at 13 000 × *g* for 15 min, the supernatant was collected, and the nucleic acid content was measured at 260 nm. Cell lysates was then loaded onto a 10% to 50% (w/v) sucrose gradient prepared in lysis buffer without Triton X‐100 and centrifuged at 4°C for 4 h at 27 500 rpm. Fractions were collected and analysed using a Gradient Station (Bio Camp, New Brunswick, Canada). Total RNA in each fraction was isolated and analysed by RT‐qPCR.

### 5.5 qPCR analysis

Samples of tumour and adjacent non‐tumorous tissues from 36 LUAD patients were analysed for levels of FTP, IGFBP3, and glyceraldehyde 3‐phosphate dehydrogenase (GAPDH) mRNA by RT‐qPCR, and levels of m^6^A at specific sites were quantified by methylated RNA immunoprecipitation followed by qPCR (MeRIP‐qPCR; see below). All primer sequences are listed in Table S2. For RT‐qPCR, total RNA was extracted using an RNA extraction kit (Sikejie, China, AC0202) and reverse‐transcribed using a cDNA synthesis kit (Sikejie, China, AG0305). qPCR reactions were conducted with mix and gene‐specific primers on a StepOnePlus Real‐Time PCR System. Relative mRNA levels were calculated using the ^ΔΔ^Ct method and normalised to expression of GAPDH.

### 5.6 Western blotting, immunohistochemistry, and immunofluorescence

For western blot analysis, protein lysates from LUAD cell lines were prepared using RIPA buffer supplemented with protease and phosphatase inhibitors, and proteins were then separated by SDS‐PAGE and transferred to 0.22 µm PVDF membranes. The membranes were blocked with 5% skim milk in 37°C and then incubated overnight at 4°C with primary antibodies against human FTO (Proteintech, China, 27226‐1‐AP), IGFBP3 (CST, USA, 64143), AKT (CST, USA, 9272), and phosphorylated (p)‐AKT (S473) (CST, USA, 4060), p‐AKT (T308) (CST, USA, 13038), GAPDH (Abcam, UK, ab181602), β‐actin (Abcam, UK, ab8227), Mettl3 (Proteintech, China, 15073‐1AP), Mettl14 (Proteintech, China, 26158‐1AP), IMP3 (Proteintech, China, 14642‐1AP), IMP2 (Proteintech, China, 22803‐1AP), IMP1 (Proteintech, China, 11601‐1AP), YTHDF1 (Proteintech, China, 17474‐1AP), YTHDF2 (Proteintech, China, 24744‐1AP), YTHDF3 (Proteintech, China, 25537‐1AP), YTHDC1 (Proteintech, China, 14392‐1AP), PATL1 (Proteintech, China, 21631‐1AP), LSM14A (Proteintech, China, 18336‐1AP), Tubulin (Proteintech, China, 80762‐1RR), Histon H3 (Proteintech, China, 17168‐1AP), PCNA (CST, USA, 13110), Annexin V (CST, USA, 8555). After washing, the membranes were incubated with horseradish peroxidase (HRP)‐conjugated secondary antibodies (Abcam, UK, ab205718) at 37°C for 1 h. Protein bands were visualised using an enhanced chemiluminescence detection kit (Yamei, China) and quantified by densitometry using a Windows system with ImageJ software.

Immunohistochemistry (IHC) was performed on formalin‐fixed paraffin‐embedded tissue sections. After deparaffinisation and rehydration, tissue sections were incubated overnight at 4°C with primary antibodies against FTO or IGFBP3 and then washed and incubated with HRP‐conjugated secondary antibodies. Colour development was achieved using diaminobenzidine chromogen, and the sections were counterstained with haematoxylin. Sections were visualised and images were captured using a light microscope. The intensity of staining was quantified using ImageJ software to assess relative protein expression levels.

For immunofluorescence (IF) staining, cells were fixed with 4% paraformaldehyde, permeabilised with 0.1% Triton X‐100, blocked with 5% bovine serum albumin, and incubated overnight at 4°C with primary antibodies against Ki67 (CST, USA, 9129) or GFP (Proteintech, China, 50430–2AP). The cells were then washed, incubated with Alexa Fluor‐conjugated secondary antibodies for 1 h at room temperature, and stained with 4′,6‐diamidino‐2‐phenylindole to visualise nuclei. Images were captured using a confocal fluorescence microscope. Co‐localisation studies with GFP‐LSM14A or other markers for P‐bodies were also conducted to assess mRNA or protein localisation within specific cellular compartments.

### 5.7 m^6^A quantification

The dot blot assay involves applying 2 µL nucleic acid samples onto a nitrocellulose membrane, allowing the spots to dry. The membranes were was left to the UV hinge in the instrument for 30 min and blocked with 5% skim milk in 37°C and then incubated overnight at 4°C with primary antibodies against m^6^A (Proteintech, China, 68055‐1Ig). After washing, the membranes were incubated with horseradish peroxidase (HRP)‐conjugated secondary antibodies at 37°C for 1 h. The subsequent methods were the same as those used for western blot analysis.

m^6^A quantification based on nucleic acid modification mass spectrometry was conducted in accordance with following procedures. 1 µg of RNA sample was mixed with buffer, S1 nuclease, phosphodiesterase, and alkaline phosphatase, and incubated at 37°C to enzymatically digest the RNA completely into nucleosides. After digestion, the samples were extracted with chloroform, and the aqueous phase was collected. The resulting solution was transferred to an autosampler vial for LC‐ESI‐MS/MS analysis. The analysis was performed using a Waters ACQUITY UPLC HSS T3 C18 column (1.8 µm, 100 mm × 2.1 mm i.d.). The mobile phases consisted of phase A (ultrapure water with 2 mM ammonium bicarbonate) and phase B (methanol with 2 mM ammonium bicarbonate). The gradient elution program was as follows: 0 min, A/B = 95:5 (v/v); 1.0 min, A/B = 95:5 (v/v); 9.0 min, A/B = 5:95 (v/v); 11.0 min, A/B = 95:5 (v/v); 11.1 min, A/B = 95:5 (v/v); 14.0 min, A/B = 95:5 (v/v). The flow rate was set to 0.30 mL/min, the column temperature was maintained at 40°C, and the injection volume was 10 µL.

m^6^A quantification based on ELISA methods was performed according to the standardised protocol provided by the kit manufacturer (EpigenTek, USA, P‐9005). Briefly, A549 cells were lysed in a lysis buffer containing protease and phosphatase inhibitors to extract total RNA. The extracted RNA was then subjected to a series of dilutions to obtain appropriate concentrations for the assay. The ELISA plate was coated with a capture antibody specific to m^6^A, and the diluted RNA samples were added to the wells. After incubation, a detection antibody conjugated to horseradish peroxidase was added to bind to the captured m^6^A‐containing RNA. Substrate was then added, and the absorbance was measured at a specific wavelength using a microplate reader. The m^6^A content in the samples was calculated based on a standard curve generated from known concentrations of m^6^A‐modified RNA. The results for m^6^A were calculated using the following formula. m^6^A% = m^6^A mount (ng)/slope.

### 5.8 Proliferation and colony‐forming assays

Cell proliferation was quantified using EdU incorporation or CCK8 assays. For the CCK8 assay, transfected cells were added to 96‐well plates at 1000 cells per well and incubated at 37°C for 24, 48, or 72 h. At each time point, 10 µL/well of CCK8 reagent was added for an additional 2 h and the absorbance at 450 nm was then measured. For organoid samples, the matrix gel is first digested before the CCK8 assay. The organoids are then digested and dissociated into single‐cell suspensions. To ensure the accuracy and reproducibility of the results, the dissociated single cells are counted in advance to guarantee 200 cells per well. For colony‐forming assays, 200 cells/well were placed in 6‐well plates and cultured for at least 10 days. Cells were fixed with 4% paraformaldehyde, stained with 0.1% crystal violet, and colonies were counted under a microscope. For EdU assays, cells were cultured in appropriate medium and treated under specified conditions. EdU (5‐ethynyl‐2′‐deoxyuridine, Invitrogen, USA) was added to the culture medium at a final concentration of 10 µM, and the cells were incubated for 2 h at 37°C in a 5% CO₂ incubator to allow EdU incorporation into the DNA of proliferating cells. After incubation, cells were fixed with 4% paraformaldehyde (PFA) for 15 min at room temperature. Next, cells were permeabilised with 0.5% Triton X‐100 in PBS for 10 min. The click reaction was performed by incubating the cells with a reaction cocktail containing the azide‐conjugated fluorophore (e.g., Alexa Fluor 488 Azide, Invitrogen, USA) for 30 min at room temperature in the dark. Nuclei were counterstained with DAPI for 5 min. The fluorescence signals were captured using a fluorescence microscope to determine the percentage of proliferating cells. EdU‐positive cells were quantified by first defining a reference fluorescence intensity threshold. Cells with EdU fluorescence exceeding this threshold were classified as EdU‐positive; the remainder were classified as negative. The result is reported as the percentage (EdU‐positive ratio).

### 5.9 Transwell migration and invasion assays

To assess migration, A549 or PC9 cells were added to the upper chamber of a Transwell insert (8‐µm pore size; Corning) at 10⁴ cells/well and the bottom chamber was filled with medium supplemented with 10% FBS as a chemoattractant. After incubation at 37°C for 24 h, non‐migrated cells on the upper surface of the insert were removed with a cotton swab, and migrated cells on the lower surface of the insert were fixed with 4% paraformaldehyde and stained with 0.1% crystal violet. Cells in five randomly selected fields were counted under a microscope. To assess invasion, the procedure described above was followed except that Transwell inserts were pre‐coated with Matrigel (Corning). Migrating or invading cells were counted and the results are expressed as the average number of cells per field.

### 5.10 Wound‐healing assay

To assess migration using an independent assay, A549 or PC9 cells were cultured to 90% confluence in 6‐well plates, and a linear wound was created by scraping a sterile pipette tip across the cell monolayer. Floating cells were removed by washing with PBS, serum‐free medium was added to the adherent cells, and the plates were incubated for up to 48 h at 37°C. Images of the wound area were captured at 0, 24, and 48 h using a microscope. The wound closure rate was calculated by measuring the wound area remaining in comparison to time 0, and cell migration is expressed as the percentage wound closure at 24 and 48 h.

### 5.11 Actinomycin D and cycloheximide assay

To assess mRNA stability, cells were treated with 5 µg/mL actinomycin D (Selleck, USA, S8964) to inhibit transcription, and RNA was extracted before and at 2, 4, 6, and 8 h post‐treatment. IGFBP3 mRNA was quantified by RT‐qPCR, and the levels were normalised to time zero to determine the mRNA decay rate. To assess protein degradation efficiency, cells were treated with 100 µg/mL of cycloheximide to inhibit protein synthesis, and supernatants from cell lysis were harvested after 0, 3, 6, 9, 12, 15 h post‐treatment. Protein degradation efficiency was then analysed by western blot experiments with β‐actin as the normalised protein.^80^

### 5.12 Dual‐luciferase reporter assay

The IGFBP3 promoter was cloned into a dual‐luciferase reporter plasmid (pGL3‐basic) and co‐transfected together with a Renilla luciferase control plasmid into A549 cells. After 72 h incubation, the cells were lysed and luciferase activity was measured using a dual‐luciferase assay kit (GenePharma, China, SA‐biotin). IGFBP3‐driven firefly luciferase activity was normalised to Renilla luciferase activity to account for transfection efficiency.

### 5.13 RNA‐binding protein immunoprecipitation (RIP) assay

RIP was used to identify RNA‐binding proteins that interact with IGFBP3 mRNA. A549 and PC9 cell lysates were incubated with specific anti‐IMP3 or anti‐YTHDC1 antibodies, and immune complexes were collected using magnetic protein G beads. RNA bound to the antibodies was extracted and analysed by qPCR to quantify IGFBP3 mRNA.

### 5.14 scRNA‐seq, MeRIP‐seq, and RNA‐seq

For single‐cell RNA sequencing (scRNA‐seq), mouse lungs were harvested, dissected, and enzymatically digested with collagenase and DNase to generate single‐cell suspensions (Miltenyi Biotec, Germany, 130‐096‐730). After red blood cell lysis, the remaining cells were filtered through a 40‐µm cell strainer. Cell viability (> 85%) was confirmed by Trypan Blue staining (Thermo, USA, T10282). Single‐cell libraries were generated using Chromium Next GEM Single Cell 3ʹ Kit v3.1 (10× Genomics, PN‐1000269). scRNA‐seq was performed using the 10× Genomics Chromium method according to the manufacturer's protocols. Libraries were sequenced on an Illumina NovaSeq 6000 platform, achieving an average depth of 100 000 reads per cell.^81^ The raw data was mapped to the mouse genome mm10‐3.0.0 using cellranger‐3.1.0. The raw sequencing data was demultiplexed and converted into a gene barcode matrix using the Cell Ranger (version 2.2.0) mkfastq and count functions (10× Genomics). Further data analysis was performed in R (version 3.4.0) using Seurat (version 3). Draw the number of genes detected in each cell, the number of Unique Molecular Identifiers (UMIs), and the percentage of mitochondrial genes, and remove outliers (cells expressing fewer than 200 and more than 2500 genes) to filter out doublets (two single cells) and dead cells. The difference between UMI count and mitochondrial read percentage is regressed. Normalise the original UMI counts and transform them logarithmically. To annotate different scRNA‐seq analysis clusters, we used cell type‐specific gene signature lists from reference.^82^ The usage of cell type‐specific gene feature lists are as follows: B cells: Ms4a1, Bank1, Cd79b, Fcer2a, Pax5, Cd79a, Tcl1, Stap1, Fcrl5, Pou2af1, Ptprc and Cd19; lymphocytes: Cd5, Ubash3a, Il7r, Itk, Cd28, Themis, Bcl11b, Cd3d, Cd3e and Cd3g; DC: Itgax, Cst3 and Cd74; migDC: Ccr7, Ccl22 and Fscn1;cDC1: Itgae, Clec9a, Ccl17 and Xcr1; pDC: Bst2, Ly6d, Siglech and Tcf4; Neutrophils: Pglyrp1, Csf3r, Cyp4f18, and Itgam; Myeloid cells: Kit, Cd34, Ly6a, Kcnip3, Med21, Sgms2, Tfec, Itgam, and Ly6g; Monocytes: Ms4a6c, Ms4a6b, Ms4a6d, and Ly6c2; Macrophages: Adgre1, Cd68, Csf1r, Fcgr1, Cd14, C1qb, C1qa, Flt3, Cx3cr1, and Vcam1. HALLMARK enrichment analysis was performed using Molecular Signatures Database (MSigDB), available through an online service. False discovery rate (FDR)–adjusted *p* < .05, retrieving a maximum of 20 terms. Single‐sample gene set enrichment analysis (ssGSEA) was used to calculate an expression score for gene expression signatures. The method was implemented in the R package GSVA.^83^

For MeRIP‐Sequencing, total RNA was extracted from cells or tissues using TRIzol reagent, and purified RNA was fragmented to ∼100 nucleotide lengths using RNase‐free fragmentation reagents. A portion of the fragmented RNA was set aside as the input sample and the remainder was incubated with an anti‐m^6^A antibody (Proteintech, China, 68055‐1‐Ig) in IP buffer. RNA high throughput sequencing was performed by CloudSeq Biotech Inc. (Shanghai, China). Briefly, total RNA was used for removing the rRNAs with GenSeq® rRNA Removal Kit (GenSeq, Inc.). Then, the rRNA‐depleted samples were subjected to library construction with GenSeq® Low Input RNA Library Prep Kit (GenSeq, Inc.) according to the manufacturer's instructions. Libraries were controlled for quality and quantified using the BioAnalyzer 2100 system (Agilent Technologies, Inc., USA). Library sequencing was performed on sequencer with 150 bp paired‐end reads. Immune complexes were collected with protein A/G magnetic beads. After washing the beads extensively, methylated RNA was eluted from the RNA‐antibody complexes using liquid buffer. The input and anti‐m^6^A‐immunoprecipitated RNA samples were used to construct libraries using standard RNA‐seq protocols. The libraries were sequenced on an Illumina NovaSeq 6000 platform to generate 150 bp paired‐end reads. Paired‐end reads were harvested from sequencer and were quality controlled by Q30. After 3′ adaptor‐trimming and low‐quality reads removing by cutadapt software (v1.9.3), the high‐quality clean reads were aligned to the reference genome with hisat2 software (v2.0.4).^84^ Then, HTSeq software (v0.9.1) was used to get the raw count, and edgeR was used to perform normalisation,^84^ then differentially expressed mRNAs were identified by *p*‐value and fold change. GO and Pathway enrichment analysis were performed based on the differentially expressed mRNAs.

For RNA sequencing, the methodologies for RNA isolation library construction and for sequencing were similar to those described above.

### 5.15 RNA‐pulldown assay

For MeRIP‐qPCR, the MeRIP assay was conducted using a m^6^A MeRIP Kit (GenSeq, China, GS‐ET‐001A) following the manufacturer's instructions with slight modifications. Briefly, total RNA was extracted and its quality was assessed using 1% agarose gel electrophoresis. RNA samples (> 100 µg) were fragmented into ∼200 nt using the kit's fragmentation buffer at 70°C for 5 min. Then, the fragmented RNA was immunoprecipitated with m^6^A‐specific antibodies coupled to magnetic beads. After a series of washes with low‐and high‐salt buffers to remove non‐specific bindings, the RNA‐antibody‐bead complexes were eluted. Subsequently, the RNA was purified using the kit's purification protocol involving magnetic beads and buffers. Finally, the purified RNA was eluted and quantified for downstream applications such as qPCR or sequencing. The kit also included control IgG antibodies for mock IP experiments to assess background noise. The entire process was carried out using nuclease‐free materials and reagents to ensure the integrity of the RNA samples.

Biotinylated RNA probes corresponding to m^6^A‐modified sites in the IGFBP3 3′UTR were synthesised and incubated with cytoplasmic lysates, and RNA complexes were then collected using streptavidin beads. Proteins bound to RNA probes were eluted, separated by SDS‐PAGE, and analysed by western blotting as described above using anti‐IMP3 and anti‐YTHDC1 primary antibodies.

### 5.16 Fluorescence in situ hybridisation

FISH was used to visualise the subcellular localisation of IGFBP3 mRNA. The cells were then carefully fixed using 4% paraformaldehyde in PBS for 15 min at room temperature to preserve the cellular structure and stabilise the target mRNA. Next, the cells were subjected to permeabilisation with 0.5% Triton X‐100 in PBS for 10 min to enhance the penetration of the RNA probes into the cells. Fluorescently labelled RNA probes targeting IGFBP3 and ADSL mRNA were hybridised to fixed A549 cells. The probes were prepared by in vitro transcription incorporating fluorescein‐12‐UTP, ensuring high specificity and sensitivity for their respective target mRNAs. Co‐localisation with GFP‐LSM14A‐labelled P‐bodies was analysed using confocal microscopy.

### 5.17 Flow cytometry for P‐body isolation

P‐bodies were labelled with GFP‐LSM14A and isolated by FACS. Cytoplasmic extracts were sorted based on size and fluorescence intensity to distinguish GFP‐LSM14A‐positive P‐bodies from non‐P‐body particles. Utilise forward scatter (FSC) and side scatter (SSC) to detect nanoparticles (CytoFLEX nano, BECKMAN, USA). FSC correlates with particle size, while SSC reflects the internal complexity of the particles. Analyse GFP‐tagged P‐bodies by detecting their fluorescent signals. Use flow cytometry software to acquire and analyse data. Set appropriate acquisition parameters to collect sufficient nanoparticle events, ensuring statistical reliability. Analyse parameters such as nanoparticle size distribution, fluorescence intensity distribution, and percentage of positive nanoparticles through software functions like histograms and dot plots. Choose a suitable nozzle size and pressure to ensure smooth droplet formation and stable sorting. Sorted fractions were analysed for m^6^A levels and IGFBP3 mRNA enrichment.

### 5.18 Statistics

Results are presented as mean ± SD. Statistical analyses were performed using Prism software (GraphPad Software 8), and consisted of analysis of variance followed by Student's *t*‐test when comparing two experimental groups. A probability of .05 or less was considered statistically significant.

## AUTHOR CONTRIBUTIONS

L.D., J.P. and Z.Y.X. conceived and designed the study; W.H.Y, P.H. and Z.Z.Z performed the experiments; Y.L.M. acquired in vivo data; H.J.Y. and Z.Y.X. resected and offered cancer tissues from lung cancer patients; W.H.Y. and P.H. analysed the data; and W.H.Y and J.P. wrote the manuscript with contributions from all co‐authors.

## CONFLICT OF INTEREST STATEMENT

The authors declare no conflicts of interest.

## ETHICS STATEMENT

This study was approved by the Ethics Committee of Tongji hospital of Tongji University (approval number: 2024‐011). All procedures performed in studies involving human participants were in accordance with the ethical standards of the institutional and/or national research committee and with the 1964 Helsinki Declaration and its later amendments or comparable ethical standards. Informed consent was obtained from all individual participants included in the study.

## Supporting information




**(A)** Expression of FTO mRNA in 19 malignant tumours in TCGA or GEO database. FTO knockdown was demonstrated by GFP protein expression **(B)** and western blots **(C)** results, indicating that we successfully constructed a lung cancer mouse model with alveolar epithelial cell‐specific FTO knockdown.

**(A‐C)** GFP protein expression (indicating lentiviral transduction) and H&E stain confirmed the morphology of patient‐derived lung cancer organoids. Successful FTO knockdown (FTO‐kd) in these organoids was validated by qRT‐PCR (B, right panel) showing significant reduction in FTO mRNA levels compared to shNC transduced organoids (*p* < .001, *n* = 3). **(D)** Western blot and qPCR results confirmed the successfully conducted of FTO‐kd A549 and PC9 cells (*n* = 3). (E‐G) EdU (*p*<.0001, *n* = 6) and CCK8 (*p*<.0001, *n* = 10) assays and immunofluorescence of Ki67 (*p*<.0001, *n* = 6) showed that FTO‐kd significantly impaired the proliferative ability of PC9 cells. **(H)** Apoptosis assays indicated a significant increase in apoptosis in FTO‐kd PC9 cells (*p*<.0001, *n* = 6). **(I, J)** Transwell assays showed that FTO‐kd significantly suppressed the invasion **(I)** (*p*<.0001, *n* = 6) and migration **(J)** (*p*<.0001, *n* = 6) abilities of A549 and PC9 cells.

**(A, B)** ELISA‐based quantification of m^6^A and m^6^Am in FTO‐kd and FTO‐oe PC9 cells, showing trends consistent with A549 cells (*n* = 3). **(C)** Nucleic acid modification mass spectrometry analysis of RNA epigenetic marks in FTO‐kd and FTO‐oe cells (*n* = 3). Significant changes were observed in all modifications except m5C, with m^6^A showing the most pronounced variations (*n* = 3). **(D)** ELISA‐based quantification of m^6^Am in lung cancer patient tissues, showing no significant difference in m^6^Am levels (*p* = .0546, *n* = 26). **(E)** RNA‐seq analysis of FTO‐kd A549 cells revealed 656 upregulated and 495 downregulated genes (*N* = 3). **(F)** MeRIP‐seq analysis identified genes with altered m^6^A methylation following FTO‐kd: 450 genes with hypomethylation and 701 with hypermethylation. **(G, H)** MeRIP‐seq identified 8660 common peaks, along with 2908 and 6103 unique peaks in NC and FTO‐kd A549 cells, respectively, and identified 1021 hypomethylated and 2304 hypermethylated sites. **(I, J)** m^6^A peaks were enriched in coding sequences and 3′UTRs, with increased density in 3′UTRs in FTO‐kd cells. **(K)** Motif analysis of m^6^A modification sites in FTO‐kd and NC groups identified conserved motifs (DDGACU and DDACDA). **(L)** Schemas of construction patterns for partial and complete mutations in the catalytic function of FTO. **(M)** MeRIP‐qPCR confirmed that FTO‐oe A549, m^6^A modifications at sites 4–7 were significantly reduced, and this effect was abolished when FTO's catalytic activity was mutated (*n* = 3).

**(A, B)** Western blot validation the expression of IGFBP3‐kd and ‐oe in lung cancer cell lines (*n* = 3). **(C)** Differential gene expression analysis in IGFBP3‐kd cells using GSE database data. **(D, E)** KEGG pathway and Gene Set Enrichment Analysis (GSEA) revealed significant enrichment of the PI3K‐Akt pathway in IGFBP3‐kd cells. **(F)** The volcano plot of differential genes indicated that AKT1 gene was downregulated after IGFBP3 knockdown. **(G)** ELISA quantification of IGFBP3 protein in the supernatant of IGFBP3‐oe and control cells. Supernatant from IGFBP3‐oe cells contained elevated IGFBP3 levels (*p*<.0001, *n* = 10). **(H, I)** Western blot analysis showed that conditioned medium from IGFBP3‐oe cells failed to activate AKT, and purified exogenous IGFBP3 (100 nM) also did not activate AKT (*n* = 3). **(J)** Treatment with the IGF1/2R inhibitor (NVP‐NEW541, 200 nM) did not block AKT activation induced by IGFBP3‐oe (*n* = 3).

**(A)** TCGA database analysis showed a significant correlation between IMP3 expression and IGFBP3 levels in lung cancer (*r* = .2741, *p*<.001). **(B)** RNA agarose gel electrophoresis confirmed the specific binding of YTHDC1 and IMP3 to the targeted m^6^A‐modified region of IGFBP3 mRNA (229 bp). **(C, D)** Actinomycin D (Act‐D) assays revealed that FTO‐kd does not affect IGFBP3 RNA stability in A549 (*p* = .2916, *n* = 3) and PC9 (*p* = .1337, *n* = 3) cells. **(E, F)** Luciferase reporter assays with IGFBP3 promoter constructs showed no significant change in IGFBP3 transcriptional activity in FTO‐kd A549 (*p* = .2471, *n* = 3) or PC9 (*p* = .3157, *n* = 3) cells. **(G, H)** Pre‐mRNA and nascent transcript measurements using Act‐D assays showed no difference in IGFBP3 splicing rates between FTO‐kd and control cells (*n* = 3). **(I, J)** Subcellular localisation analysis of IGFBP3 mRNA showed no difference in nuclear and cytoplasmic distributions between FTO‐kd and control cells (*n* = 3). **(K, L)** qPCR analysis of nuclear and cytoplasmic RNA fractions further confirmed no effect of FTO‐kd on IGFBP3 mRNA localisation in A549 (*p* = .3828, *n* = 3) or PC9 (*p* = .2907, *n* = 3). **(M)** Western blot analysis of IGFBP3 protein stability after cycloheximide (CHX) treatment showed no significant changes in its half‐life in FTO‐kd cells (*p* = .3101, *n* = 3). **(N)** Absolute quantification of IGFBP3 transcript copy number and TE in YTHDC1‐kd and control cells (*p*>.05, *n* = 4)

**Figure S6**. IMP3 inhibits IGFBP3 mRNA translation by promoting its localisation to P‐bodies.
**(A)** ELISA analysis indicated that IMP3‐kd did not change the m^6^A between individual components of the polyribosome (*n* = 3). **(B)** There was no difference in transfection efficiency of the plasmids between two groups (*n* = 3). **(C)** IMP3‐oe enhanced the co‐localisation of IGFBP3 mRNA with P‐bodies (*p*<.0001, *n* = 3).

Supporting Information

## Data Availability

RNA‐seq, MeRIP‐seq and scRNA‐seq data generated in this study have been deposited in the Sequence Read Archive (SRA) under accession number PRJNA1196085 and PRJNA1196111.
