# Supplementary figures and images for: FTO‐mediated m6A demethylation regulates IGFBP3 expression and AKT activation through IMP3‐dependent P‐body re‐localisation in lung cancer
Source: Clin Transl Med. 2025 Jul 7;15(7):e70392. doi: 10.1002/ctm2.70392 (PMC12230637; doi:10.1002/ctm2.70392)

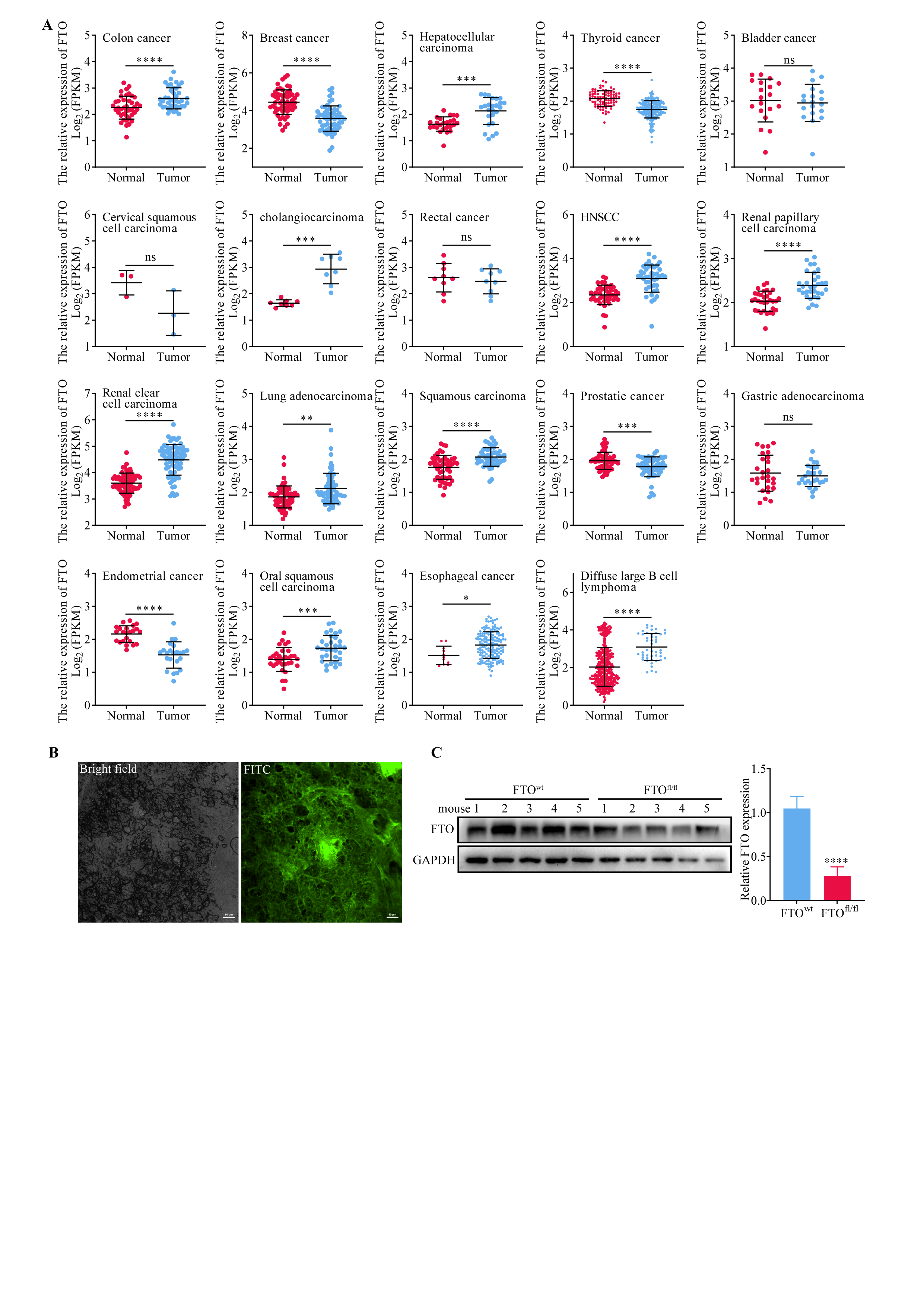

Supplement: Supplementary file 1 — (A) Expression of FTO mRNA in 19 malignant tumours in TCGA or GEO database. FTO knockdown was demonstrated by GFP protein expression (B) and western blots (C) results, indicating that we successfully constructed a lung cancer mouse model with alveolar epithelial cell‐specific FTO knockdown. [file CTM2-15-e70392-s003.tif]

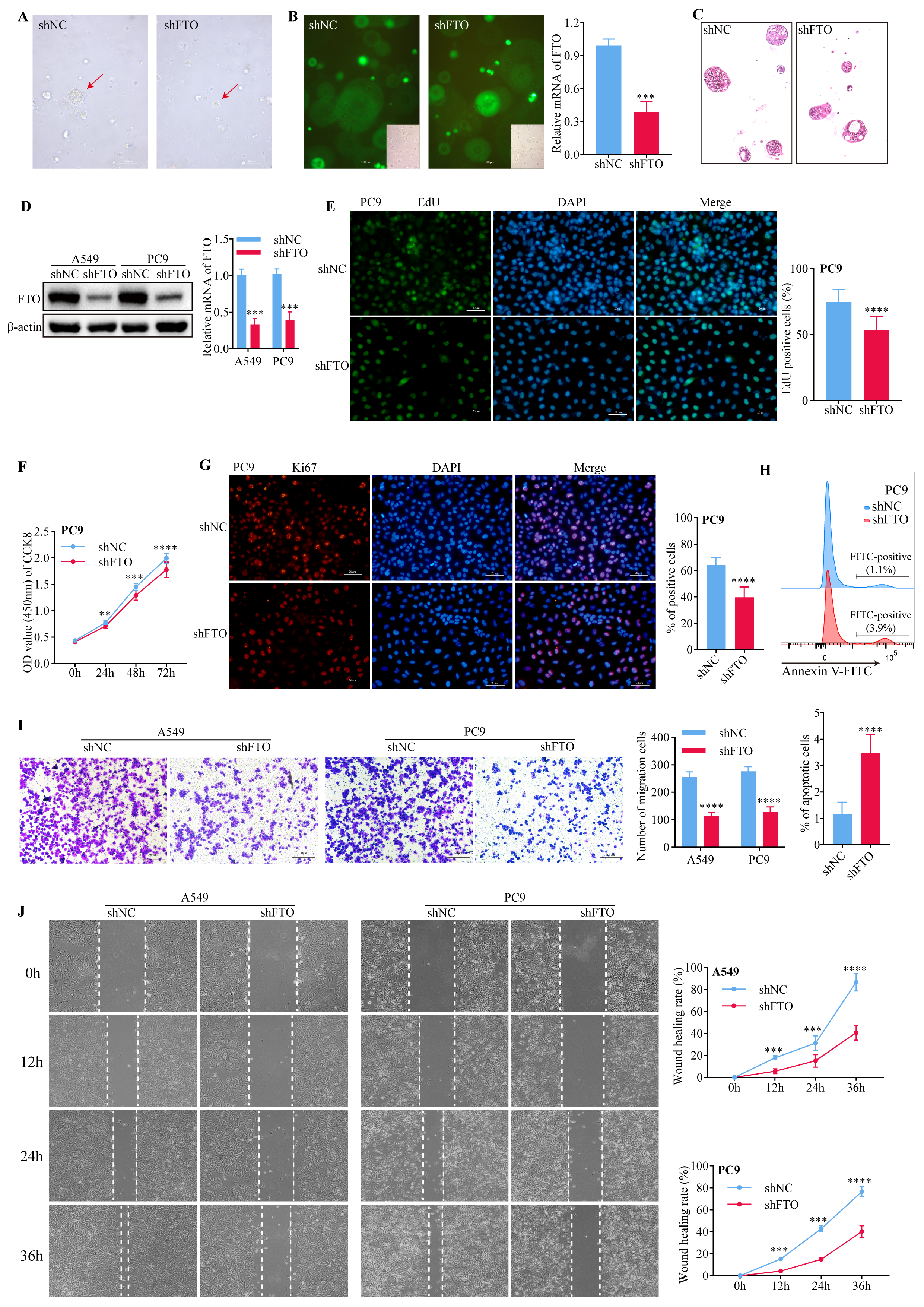

Supplement: Supplementary file 2 — (A‐C) GFP protein expression (indicating lentiviral transduction) and H&E stain confirmed the morphology of patient‐derived lung cancer organoids. Successful FTO knockdown (FTO‐kd) in these organoids was validated by qRT‐PCR (B, right panel) showing significant reduction in FTO mRNA levels compared to shNC transduced organoids (p < .001, n = 3). (D) Western blot and qPCR results confirmed the successfully conducted of FTO‐kd A549 and PC9 cells (n = 3). (E‐G) EdU (p<.0001, n = 6) and CCK8 (p<.0001, n = 10) assays and immunofluorescence of Ki67 (p<.0001, n = 6) showed that FTO‐kd significantly impaired the proliferative ability of PC9 cells. (H) Apoptosis assays indicated a significant increase in apoptosis in FTO‐kd PC9 cells (p<.0001, n = 6). (I, J) Transwell assays showed that FTO‐kd significantly suppressed the invasion (I) (p<.0001, n = 6) and migration (J) (p<.0001, n = 6) abilities of A549 and PC9 cells. [file CTM2-15-e70392-s002.tif]

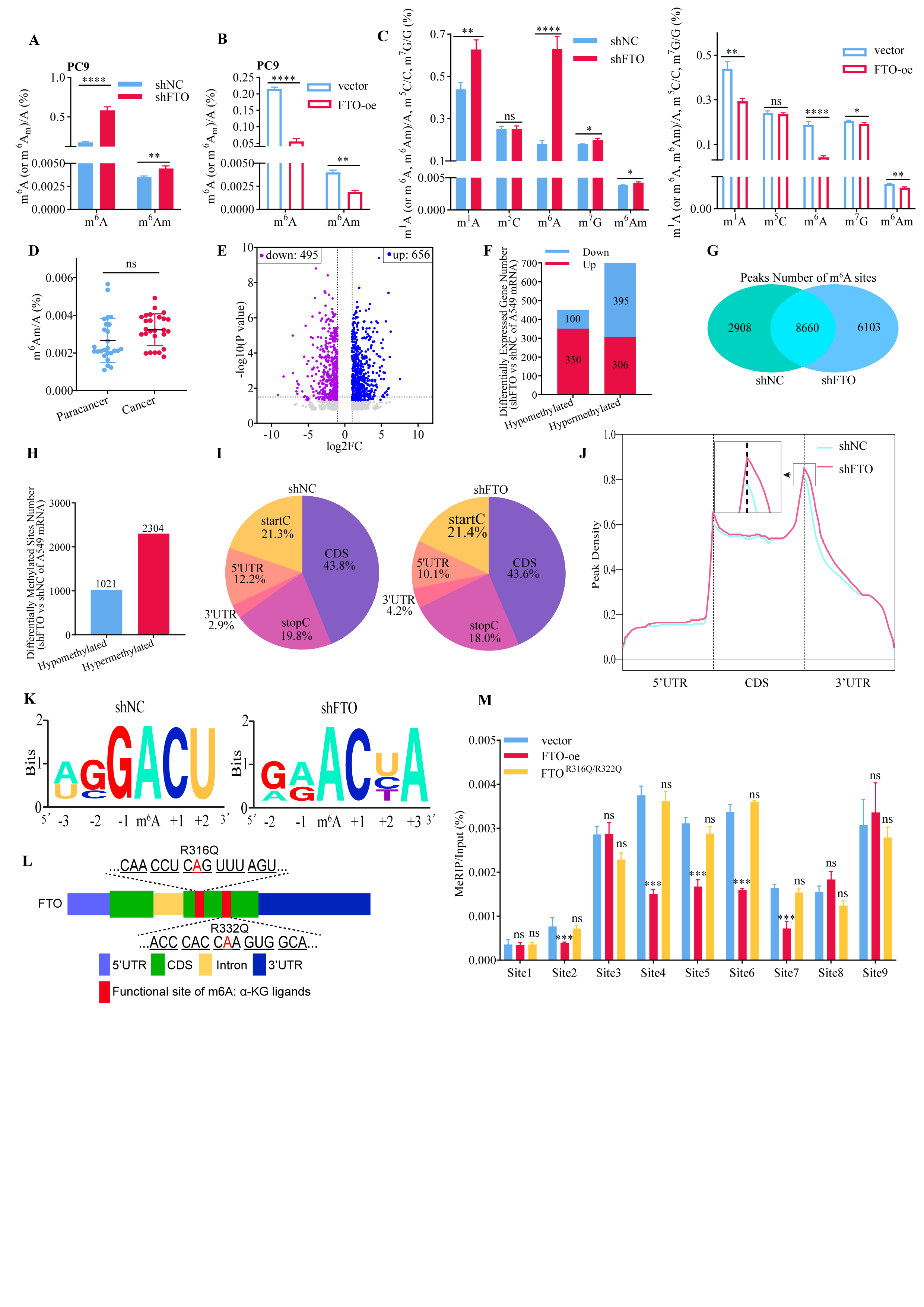

Supplement: Supplementary file 3 — (A, B) ELISA‐based quantification of m6A and m6Am in FTO‐kd and FTO‐oe PC9 cells, showing trends consistent with A549 cells (n = 3). (C) Nucleic acid modification mass spectrometry analysis of RNA epigenetic marks in FTO‐kd and FTO‐oe cells (n = 3). Significant changes were observed in all modifications except m5C, with m6A showing the most pronounced variations (n = 3). (D) ELISA‐based quantification of m6Am in lung cancer patient tissues, showing no significant difference in m6Am levels (p = .0546, n = 26). (E) RNA‐seq analysis of FTO‐kd A549 cells revealed 656 upregulated and 495 downregulated genes (N = 3). (F) MeRIP‐seq analysis identified genes with altered m6A methylation following FTO‐kd: 450 genes with hypomethylation and 701 with hypermethylation. (G, H) MeRIP‐seq identified 8660 common peaks, along with 2908 and 6103 unique peaks in NC and FTO‐kd A549 cells, respectively, and identified 1021 hypomethylated and 2304 hypermethylated sites. (I, J) m6A peaks were enriched in coding sequences and 3′UTRs, with increased density in 3′UTRs in FTO‐kd cells. (K) Motif analysis of m6A modification sites in FTO‐kd and NC groups identified conserved motifs (DDGACU and DDACDA). (L) Schemas of construction patterns for partial and complete mutations in the catalytic function of FTO. (M) MeRIP‐qPCR confirmed that FTO‐oe A549, m6A modifications at sites 4–7 were significantly reduced, and this effect was abolished when FTO's catalytic activity was mutated (n = 3). [file CTM2-15-e70392-s007.tif]

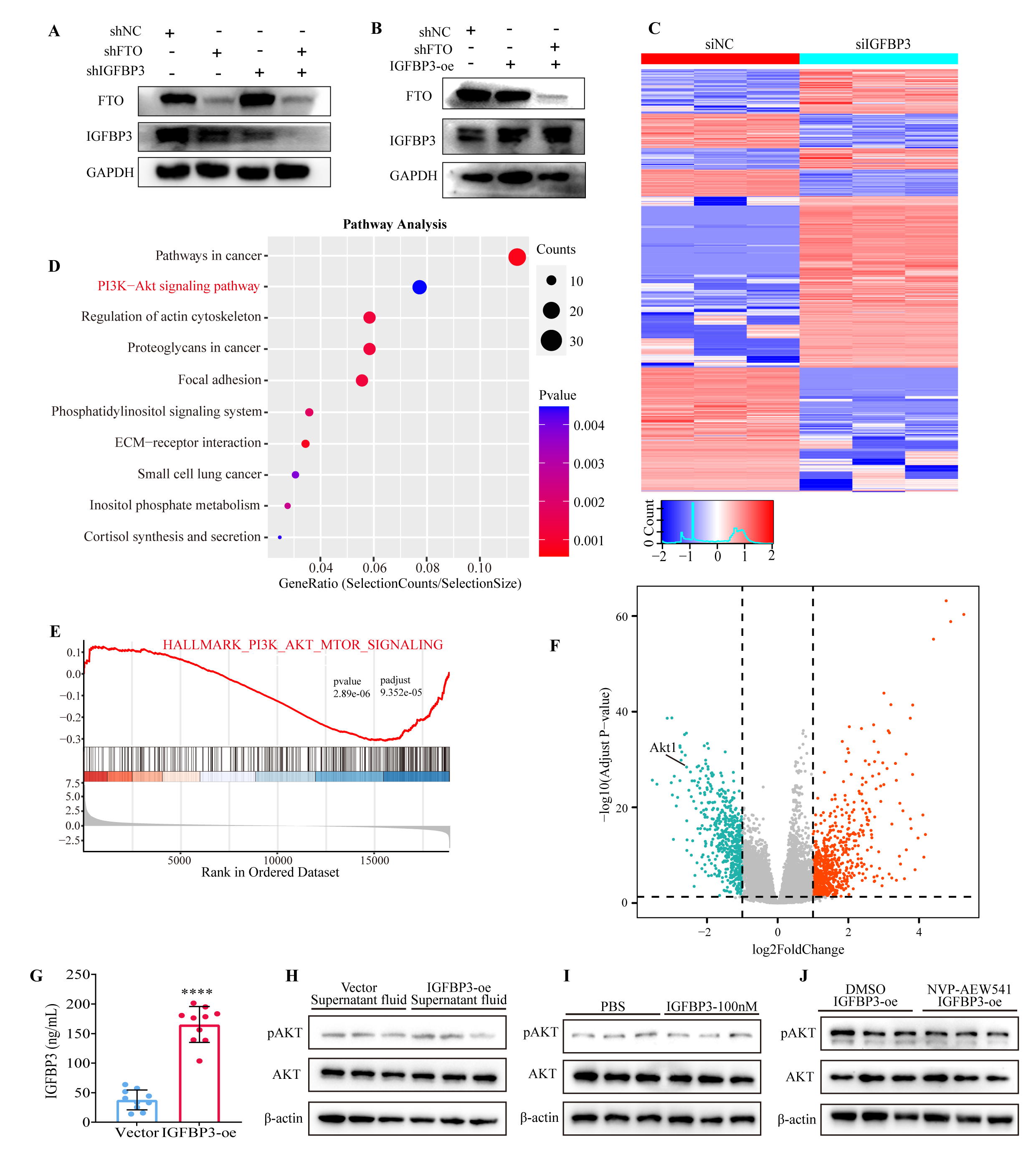

Supplement: Supplementary file 4 — (A, B) Western blot validation the expression of IGFBP3‐kd and ‐oe in lung cancer cell lines (n = 3). (C) Differential gene expression analysis in IGFBP3‐kd cells using GSE database data. (D, E) KEGG pathway and Gene Set Enrichment Analysis (GSEA) revealed significant enrichment of the PI3K‐Akt pathway in IGFBP3‐kd cells. (F) The volcano plot of differential genes indicated that AKT1 gene was downregulated after IGFBP3 knockdown. (G) ELISA quantification of IGFBP3 protein in the supernatant of IGFBP3‐oe and control cells. Supernatant from IGFBP3‐oe cells contained elevated IGFBP3 levels (p<.0001, n = 10). (H, I) Western blot analysis showed that conditioned medium from IGFBP3‐oe cells failed to activate AKT, and purified exogenous IGFBP3 (100 nM) also did not activate AKT (n = 3). (J) Treatment with the IGF1/2R inhibitor (NVP‐NEW541, 200 nM) did not block AKT activation induced by IGFBP3‐oe (n = 3). [file CTM2-15-e70392-s001.tif]

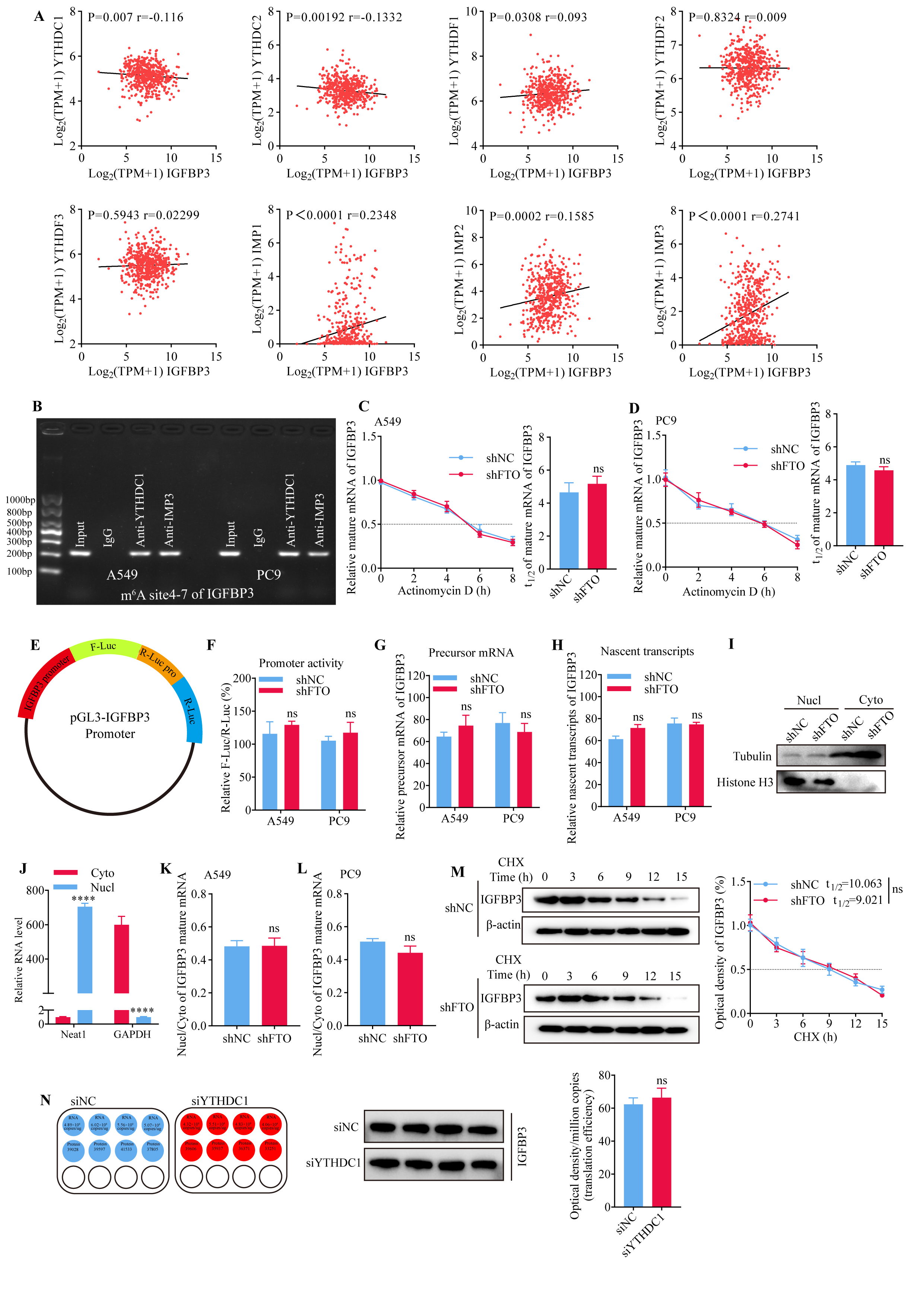

Supplement: Supplementary file 5 — (A) TCGA database analysis showed a significant correlation between IMP3 expression and IGFBP3 levels in lung cancer (r = .2741, p<.001). (B) RNA agarose gel electrophoresis confirmed the specific binding of YTHDC1 and IMP3 to the targeted m6A‐modified region of IGFBP3 mRNA (229 bp). (C, D) Actinomycin D (Act‐D) assays revealed that FTO‐kd does not affect IGFBP3 RNA stability in A549 (p = .2916, n = 3) and PC9 (p = .1337, n = 3) cells. (E, F) Luciferase reporter assays with IGFBP3 promoter constructs showed no significant change in IGFBP3 transcriptional activity in FTO‐kd A549 (p = .2471, n = 3) or PC9 (p = .3157, n = 3) cells. (G, H) Pre‐mRNA and nascent transcript measurements using Act‐D assays showed no difference in IGFBP3 splicing rates between FTO‐kd and control cells (n = 3). (I, J) Subcellular localisation analysis of IGFBP3 mRNA showed no difference in nuclear and cytoplasmic distributions between FTO‐kd and control cells (n = 3). (K, L) qPCR analysis of nuclear and cytoplasmic RNA fractions further confirmed no effect of FTO‐kd on IGFBP3 mRNA localisation in A549 (p = .3828, n = 3) or PC9 (p = .2907, n = 3). (M) Western blot analysis of IGFBP3 protein stability after cycloheximide (CHX) treatment showed no significant changes in its half‐life in FTO‐kd cells (p = .3101, n = 3). (N) Absolute quantification of IGFBP3 transcript copy number and TE in YTHDC1‐kd and control cells (p>.05, n = 4) [file CTM2-15-e70392-s006.tif]

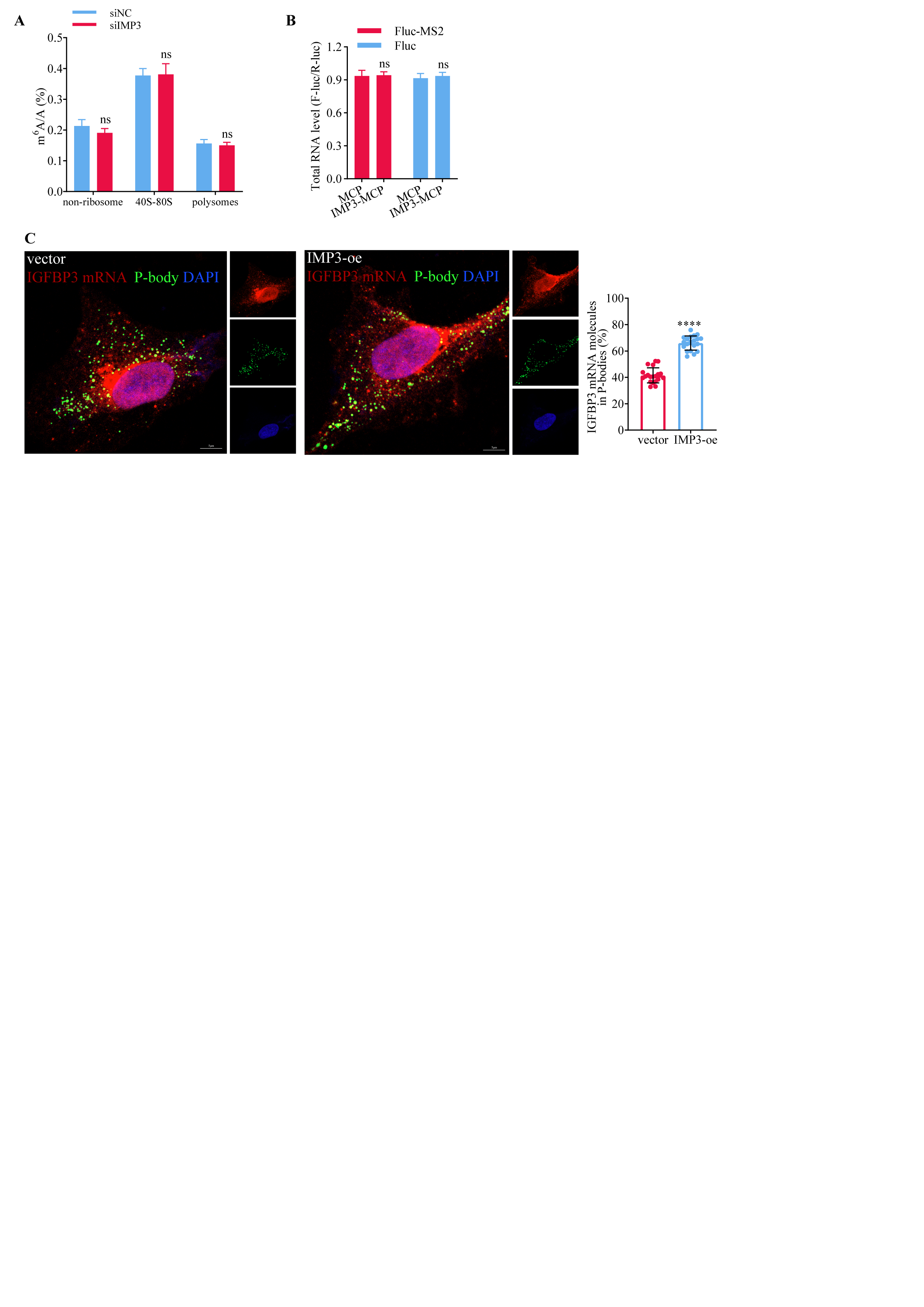

Supplement: Supplementary file 6 — Figure S6. IMP3 inhibits IGFBP3 mRNA translation by promoting its localisation to P‐bodies. (A) ELISA analysis indicated that IMP3‐kd did not change the m6A between individual components of the polyribosome (n = 3). (B) There was no difference in transfection efficiency of the plasmids between two groups (n = 3). (C) IMP3‐oe enhanced the co‐localisation of IGFBP3 mRNA with P‐bodies (p<.0001, n = 3). [file CTM2-15-e70392-s005.tif]
