# Supplementary material for: FTO‐mediated m6A demethylation regulates IGFBP3 expression and AKT activation through IMP3‐dependent P‐body re‐localisation in lung cancer
Source: Clin Transl Med. 2025 Jul 7;15(7):e70392. doi: 10.1002/ctm2.70392 (PMC12230637; doi:10.1002/ctm2.70392)
Supplement: Supplementary file 7 — Supporting Information [file CTM2-15-e70392-s004.docx]

Table S1

| Gene | Target | Sequence (5’-3’) |
| --- | --- | --- |
| FTO | shRNA | TTTGTTATA**TCCCATTAGGTGCCCATAT**TTAAAAATTGGGA |
| IGFBP3 | shRNA | CTCCTCCGAG**TCCAAGCGGGAGACAGAATAT**GGTCCCTGCC |
| IMP3 | shRNA | AAAGGATTCG**GAAACTTCAGATACGAAATAT**CCCGCCTCAT |
| METTL3 | siRNA | GCTGCACTTCAGACGAATTAT |
| METTL14 | siRNA | GAACCTGAAATTGGCAATATA |
| IMP3 | siRNA | GAAACTTCAGATACGAAATAT |
| YTHDC1 | siRNA | CACCAGAGACCAGGGTATTTA |

| Gene | Primer | Sequence (5’-3’) |
| --- | --- | --- |
| FTO | F | ACTTGGCTCCCTTATCTGACC |
|  | R | TGTGCAGTGTGAGAAAGGCTT |
| IGFBP3 | F | GCCAGCTCCAGGAAATGCTA |
|  | R | GCCAGCTCCAGGAAATGCTA |
| IGFBP3 pre-mRNA | F | TCAACGCTAGTGCCGTCAG |
|  | R | ACGCAGCGCACCTGG |
| GAPDH | F | TGATGACATCAAGAAGGTGG |
|  | R | TTGTCATACCAGGAAATGAGC |
| m6A site1 | F | GACTCTGCTGGTGCTGCTC |
|  | R | CACCACGGGACCCAAGC |
| m6A site2 | F | AGTGAGTCGGAGGAAGACCG |
|  | R | GGGGTGGAACTTGGGATCAG |
| m6A site3 | F | GTGGAGCTCAAATATGCCTT |
|  | R | TAAATCGAGGCTGTAGCCAG |
| m6A site4 | F | AGAGGTTTTTGAAATGCCTATGGT |
|  | R | TGCTGACTACTGGAAAGTGAAA |
| m6A site5 | F | TCCAGTAGTCAGCAAAGAGCA |
|  | R | CATGTGGTGAGCATTCCACG |
| m6A site6 | F | AGCACAGCACCCAGACTTC |
|  | R | TCAGTGGTCGGCCGCTTC |
| m6A site7 | F | CAGTGCGCACAGGCTTTATC |
|  | R | GGATCCACGCCCTTGTTTCA |
| m6A site8 | F | GTGGATCCCTCAACCAAGAAGA |
|  | R | TTCTCCAATAGTCCCCAAGCA |
| m6A site9 | F | TCTGGCAAAGTCAGGCTCAG |
|  | R | GAGAGCTCTATGCAGCGTGT |

Table S2
